# Straightforward Access to Thiocyanates via Dealkylative Cyanation of Sulfoxides

**DOI:** 10.1021/acs.orglett.1c00460

**Published:** 2021-03-16

**Authors:** Uroš Todorović, Immo Klose, Nuno Maulide

**Affiliations:** Institute of Organic Chemistry, University of Vienna, Währinger Strasse 38, 1090 Vienna, Austria

## Abstract

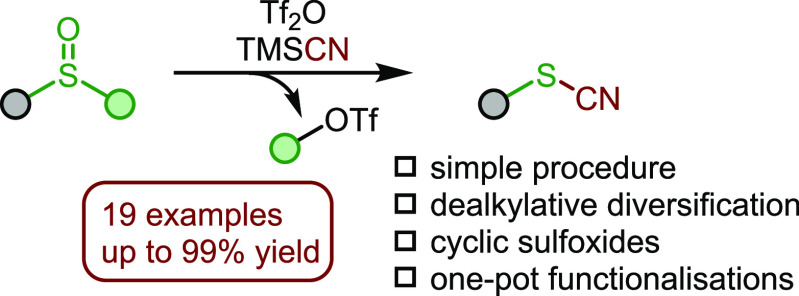

Thiocyanates, versatile building blocks in organic synthesis, are shown to be easily accessible via an interrupted Pummerer reaction of sulfoxides. This facile dealkylative functionalization proceeds under mild conditions through electrophilic activation of the sulfoxide partner. The resulting thiocyanate itself can serve as a handle for diversification in a straightforward one-pot procedure.

Thiocyanates are an important compound family widely encountered in medicinal chemistry and natural products, and they constitute versatile synthetic handles.^1,2^ Their ability to function as electrophilic components either on sulfur or on carbon renders them especially attractive intermediates.^3^

The preparation of thiocyanates mainly relies on nucleophilic substitution or coupling reactions using the thiocyanate anion (Scheme 1a).^4^ Alternative, less common methods include electrophilic thiocyanations, nucleophilic or electrophilic cyanation of suitable sulfur species, or radical processes.^5^ In 2015, Shi and coworkers reported the union of a sulfide, a sulfur species that possesses neither an acidic proton nor a designated leaving group, with a modified version of Stang’s reagent.^6^ The thiocyanate products are thus formed through oxidative cyanation followed by dealkylation (Scheme 1b). In 2019, Yang *et al.* showed that the same transformation could be achieved without the hypervalent iodine reagent, employing Selectfluor as an oxidant alongside a cyanide source.^7^

**Scheme 1:**
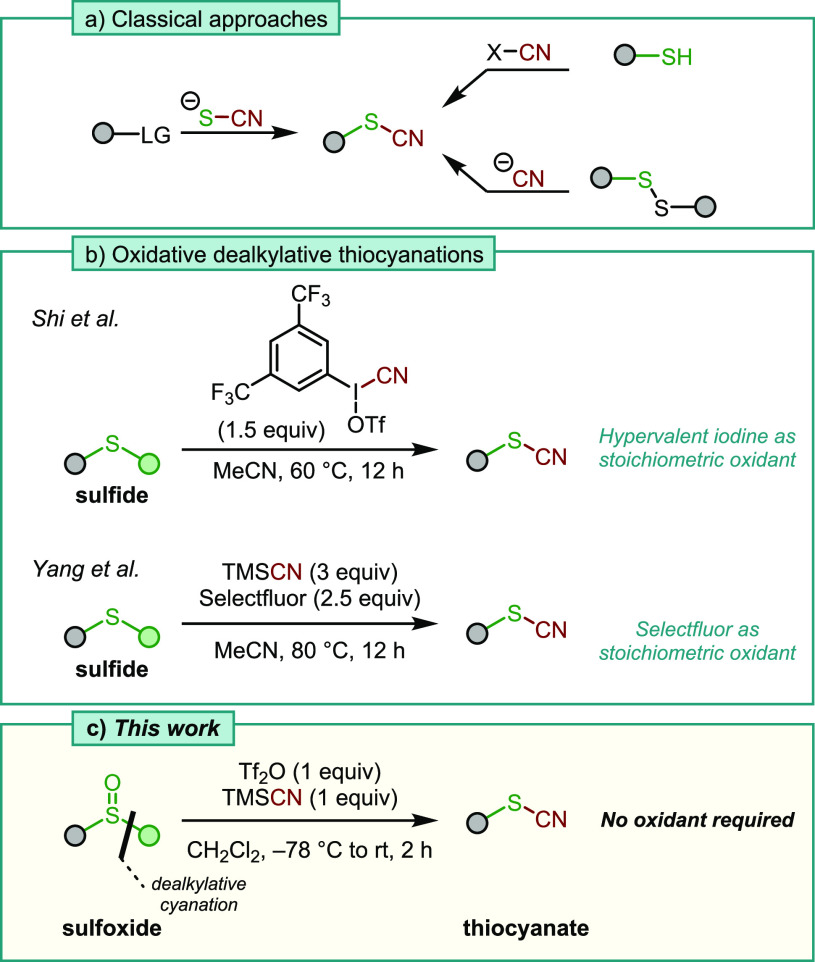
(a) Overview of Classical Thiocyanate Syntheses; (b) Oxidative Dealkylative Thiocyanations from Sulfides Using an Excess of Oxidants; and (c) Dealkylative Cyanation of Sulfoxides

In this context, we speculated that the use of strong oxidants might be avoided if one were to employ a *sulfoxide* as a reactant rather than its sulfide counterpart. Such a transformation would also further expand the toolbox for sulfoxide-mediated transformations, a field that has seen rapid development in recent years.^8^ Apart from their use as directing groups^9^ and ligands,^10^ sulfoxides are known for their propensity toward activation with electrophilic reagents, creating highly reactive species that can be synthetically exploited in a variety of reactions.^11,12^ In several of those reports, the sulfur residue that remains in the final products is often an afterthought from a synthetic point of view. Herein we report an operationally simple dealkylative conversion of sulfoxides into thiocyanates as well as related transformations (Scheme 1c).

In an initial experiment, stoichiometric trimethylsilyl cyanide was added to a mixture of *p*-tolylmethylsulfoxide **1a** and triflic anhydride (i.e., an electrophilically *activated* sulfoxide) at low temperature (Scheme 2). Satisfyingly, this resulted in a clean conversion into *p*-tolylthiocyanate **2a**, which was isolated in 91% yield. Further changes to the temperature, time of addition, and order of addition did not improve the outcome, leading us directly to the exploration of the generality of this protocol with different sulfoxides.

**Scheme 2:**
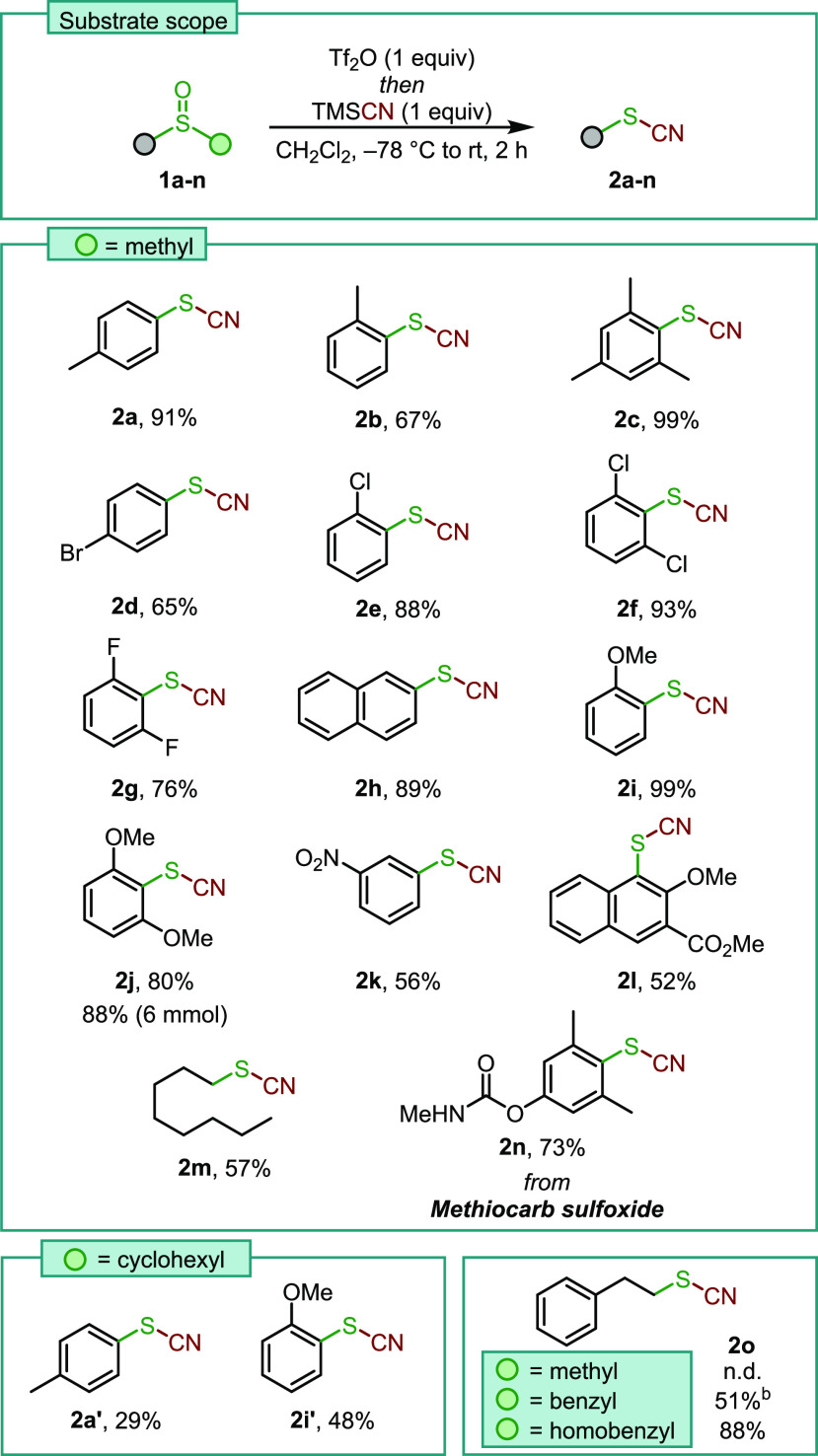
Substrate Scope for the Dealkylative Cyanation of Sulfoxides Reactions were performed on a 0.1 to 0.5 mmol scale in CH_2_Cl_2_ (0.1 M). Yield determined by ^1^H NMR using an internal standard. n.d. = not detected.

The desired thiocyanates were generally obtained in good to excellent yields. In particular, hindered mesitylsulfoxide **1c** allowed the isolation of the respective thiocyanate in excellent 99% yield. Different halide substitution patterns were also well tolerated (**2d**–**2g**), and the reaction worked well with the extended aromatic system of **2h**. Electron-rich sulfoxides furnished the respective aryl thiocyanates cleanly in high yields, whereas aryl sulfoxides bearing electron-withdrawing groups afforded the respective thiocyanates **2k**,**2l** with lower efficiency. Furthermore, it was intriguing to investigate the regioselectivity of the dealkylation step for a dialkylsulfoxide: In this event, octylmethylsulfoxide **1m** was selectively dealkylated at the more sterically accessible methyl substituent to give thiocyanate **2m**. Notably, the reaction also proceeded smoothly on Methiocarb sulfoxide **1n**, a pesticide metabolite, to give the thiocyanated derivative in 73% yield. Next, we investigated the effect of variation of the alkyl substituent. As might be expected from a dealkylative process, lower yields are observed with sulfoxides carrying secondary alkyl moieties, a clear indicator of the more challenging C–S bond-breaking event in these cases (**2a′** and **2i′**). Interestingly, preferential dealkylation of the homobenzyl substituent was observed over a methyl substituent.^13^ The formation of homobenzyl thiocyanate **2o** was achieved by changing the methyl for a benzyl and a homobenzyl substitutent, leading to a 51% yield and quantitative (88% isolated yield) formation of **2o**, respectively. Finally, the robustness and scalability of our methodology was demonstrated by subjecting **1j** to the standard conditions, delivering 1.03 g of **2j** (88%) without the need for column chromatography.

Our proposed mechanism is outlined in Scheme 3a. After the electrophilic activation of the sulfoxide to intermediate **I**_**1**_, the addition of TMSCN forms cyanosulfonium triflate **I**_**2**_.^14^ This species is readily dealkylated by the counteranion to reveal thiocyanate and the alkyl triflate.^6,7^ To provide further evidence of this mechanism, we subjected cyclic sulfoxide **1p** to the reaction conditions (Scheme 3b). To our delight, the ring-opened product was obtained in almost quantitative yield, bearing the expected triflate group on the alkyl chain.

**Scheme 3:**
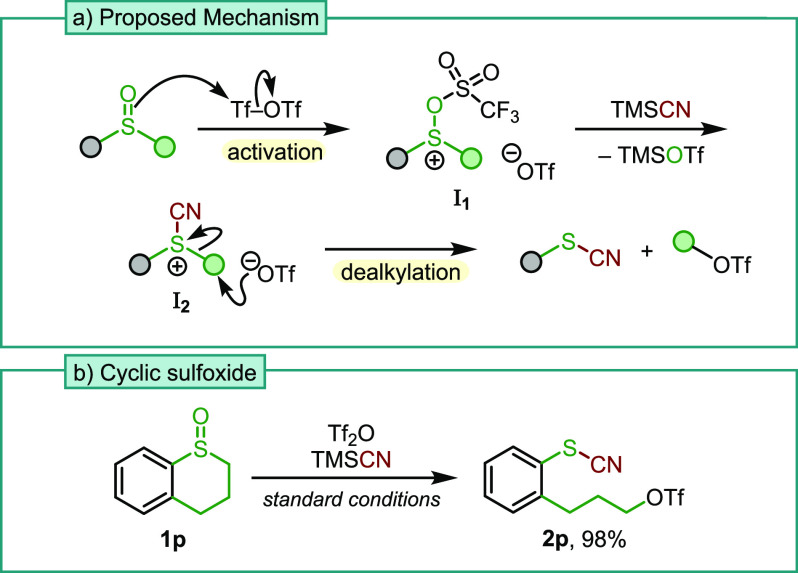
(a) Proposed Mechanism and (b) Reaction with Cyclic Sulfoxide

The simple reaction setup of this transformation led us to investigate the possibility of functionalizing the sulfoxide directly into diverse substituents in a one-pot fashion (Scheme 4). To this end, the crude reaction mixture of the dealkylative cyanation was exposed to a range of conditions. For instance, the addition of a solution of lithium alkynylide in THF smoothly afforded thioalkyne **3** in 80% isolated yield.^15^ Similarly, the addition of Ruppert’s reagent (TMSCF_3_) and TBAF was successful to afford trifluoromethyl sulfide **4** in one pot.^16^ Lastly, sulfonyl cyanide **5** could be obtained by an oxidation protocol developed by Landais and coworkers using a combination of hydrogen peroxide and trifluoroacetic anhydride (TFAA) in dichloromethane.^17^ These transformations highlight another advantage of the method presented herein, namely, the relatively clean formation of the thiocyanate even before workup of the reaction mixture, which enables a range of useful downstream processes in cases where the thiocyanate might not be the desired end product.

**Scheme 4:**
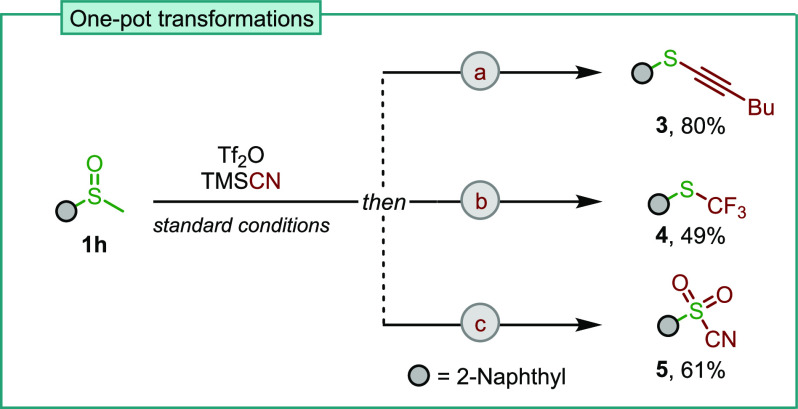
One-Pot Dealkylative Transformations of Sulfoxide Initial dealkylative cyanations were performed as in Scheme 2, which were followed by one-pot transformations: (a) 1-hexyne, *n*-BuLi, 0 °C to rt, 15 h; (b) TMSCF_3_, TBAF, 0 °C to rt, 15 h; (c) H_2_O_2_, TFAA, 40 °C, 16 h. See the Supporting Information for details.

In summary, we have presented a straightforward method to convert sulfoxides into thiocyanates with concomitant C–S bond cleavage. This dealkylative cyanation is tolerant of a broad range of substituents, including electron-rich and -deficient aryl moieties as well as aliphatic sulfoxides. Furthermore, several one-pot transformations demonstrate the synthetic utility of the protocol. We believe this method shall find broad applicability in thiocyanate chemistry.

## Supporting Information Available

Synthetic procedures and full characterization for all new compounds and NMR spectra (PDF)
