# Supplementary material for: Straightforward Access to Thiocyanates via Dealkylative Cyanation of Sulfoxides
Source: Org Lett. 2021 Mar 16;23(7):2510–3. doi: 10.1021/acs.orglett.1c00460 (PMC8022320; doi:10.1021/acs.orglett.1c00460)
Supplement: Supplementary file 1 — ol1c00460_si_001.pdf [file ol1c00460_si_001.pdf]

Straightforward Access to Thiocyanates *via* Dealkylative Cyanation of Sulfoxides

Uroš Todorović,[a]+ Immo Klose,[a]+ and Nuno Maulide*[a]

[a] Institute of Organic Chemistry, University of Vienna, Währinger Straße 38, 1090 Vienna (Austria)
E-Mail: nuno.maulide@univie.ac.at, Homepage: http://maulide.univie.ac.at

Table of Contents

1. General Information 2
2. Experimental Procedures 3
 2.1. Synthesis of Starting Materials 3
 2.2. Dealkylative cyanation 4
  2.2.1. General Procedure: Dealkylative Cyanation of Sulfoxides 4
 2.3. One-pot transformations 14
3. NMR Spectra 19
4. References 41

1. General Information

Unless otherwise stated, all glassware was flame-dried before use and all reactions were performed under an atmosphere of argon. All solvents were distilled from appropriate drying agents prior to use. All reagents were used as received from commercial suppliers unless otherwise stated. Trifluoromethanesulfonic anhydride (Tf_2O) was distilled over P_4O_{10} prior to use and stored under inert atmosphere in the fridge for a maximum of roughly 3 weeks.[1] Reaction progress was monitored by thin layer chromatography (TLC) performed on aluminum plates coated with silica gel F254 with 0.2 mm thickness. Chromatograms were visualized by fluorescence quenching with UV light at 254 nm or by staining using potassium permanganate. Flash column chromatography was performed using silica gel 60 (230-400 mesh, Merck and co.). Neat infrared spectra were recorded using a Perkin-Elmer Spectrum 100 FT-IR spectrometer. Wavenumbers (ν_{max}) are reported in cm^{-1}. Mass spectra were obtained using a Bruker maXis UHR-TOF spectrometer with electrospray ionization (ESI) and a Qq-TOF mass analyzer. In several cases, the exact mass was not detected with ESI ionization. These were measured using an Agilent 7200B GC/Q-TOF Spectrometer with electron impact (EI) ionization method and a Q-TOF mass analyzer. The fragmentation pattern for the most significant fragmentations is reported with the relative intensity of the detected mass in percent denoted in parenthesis. Melting points were determined on a capillary apparatus and are uncorrected.

All ^{1}H NMR and ^{13}C NMR spectra were recorded using a Bruker AV-400, AV-500 or AV-600 spectrometer at 300K. Chemical shifts are given in parts per million (ppm, δ), referenced to the solvent peak of $CDCl_3$, defined at δ = 7.26 ppm (^{1}H-NMR) and δ = 77.16 (^{13}C-NMR). Coupling constants are quoted in Hz (*J*). ^{1}H NMR splitting patterns are designated as singlet (s), doublet (d), triplet (t), quartet (q) as they appeared in the spectrum. If the appearance of a signal differs from the expected splitting pattern, the observed pattern is designated as apparent (app). Splitting patterns that could not be interpreted or easily visualized are designated as multiplet (m) or broad (br).

2. Experimental Procedures

2.1. Synthesis of Starting Materials

Sulfoxide **1n** was received from commercial supplier. Sulfoxides **1a**,[2] **1a'**,[3] **1b**,[4] **1c**,[4] **1d**,[2] **1e**,[5] **1f**,[5] **1g**,[5] **1h**,[6] **1i**,[4] **1i'**,[4] **1j**,[4] **1m**,[7] **1o**,[8] **1o'**,[9] **1o''**[10], **1p**[11] are known in the literature and were obtained by oxidation from the corresponding sulfides.

Sulfoxide **1k**[12] was obtained from 3-nitroaniline via a two-step literature procedure.[13]

methyl 3-methoxy-4-(methylsulfinyl)-2-naphthoate (1l)

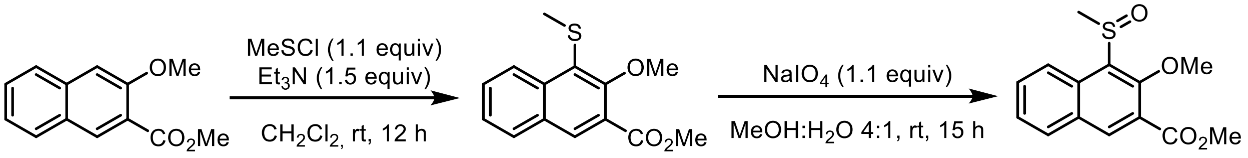

S1 **S2** **1l**

A solution of sulfenyl chloride (freshly made from dimethyl disulfide and sulfuryl chloride,[14] 0.5 M in CH_2Cl_2, 4.4 mL, 2.2 mmol, 1.1 equiv.) was added to a solution of methyl 3-methoxy-2-naphthoate **S1** (432 mg, 2.0 mmol, 1.0 equiv.)[15] and triethylamine (0.42 mL, 3.0 mmol, 1.5 equiv.) in CH_2Cl_2 (20 mL, 0.1 M) and the reaction was stirred for 12 h at ambient temperature (23 °C). Water was added (20 mL) and the reaction mixture was extracted with CH_2Cl_2 (2 × 10 ml). The combined organic phases were dried over anhydrous magnesium sulfate, filtered and the solvent removed under reduced pressure. The crude residue was purified by flash column chromatography (SiO_2, heptane/ethyl acetate) affording methyl sulfide **S2** as a mixture with the starting material in a ratio of 1.15:1 (317.2 mg, 53% purity).

Methyl 3-methoxy-4-(methylthio)-2-naphthoate **S2** (53% purity, 247 mg, 0.50 mmol, 1.0 equiv.) was dissolved in methanol (10 mL, 0.05 M). A solution of sodium periodate in water (118.0 mg in 2 mL H_2O, 0.55 mmol, 1.1 equiv.) was added and the reaction was stirred for 15 h at ambient temperature (23 °C). The precipitate was filtrated and the filtrate was evaporated *in vacuo* to about 20% of its volume. Ethyl acetate (5 mL) and H_2O (5 mL) were added and the reaction mixture was extracted with ethyl acetate (2 x 5 mL). The combined organic phases were dried

over anhydrous magnesium sulfate, filtered and the solvent removed under reduced pressure. The crude residue was purified by flash column chromatography (SiO_2, heptane/ethyl acetate) affording the title compound (114.5 mg, 26% over 2 steps) as a yellow crystalline solid.

^{1}H NMR (400 MHz, $CDCl_3$): δ 9.28 (dd, *J* = 8.7, 0.8 Hz, 1H), 8.53 (s, 1H), 7.97 – 7.88 (m, 1H), 7.66 (ddd, *J* = 8.6, 6.9, 1.4 Hz, 1H), 7.54 (ddd, *J* = 8.1, 6.9, 1.1 Hz, 1H), 4.00 (s, 3H), 3.98 (s, 3H), 3.11 (s, 3H).

^{13}C NMR (101 MHz, $CDCl_3$): δ 165.4, 154.8, 137.7, 133.6, 132.0, 130.7, 130.3, 129.8, 126.7, 123.2, 123.1, 64.0, 52.8, 40.7.

IR (neat) ν_{max}: 2943, 1721, 1445, 1439, 1209, 1077, 1054, 998, 954, 793, 756 cm^{-1}.

HRMS (ESI$^+$): exact mass calculated for $[M+Na]^+$ ($C_{14}H_{14}O_4SNa$) *m/z* 301.0505, found *m/z* 301.0502.

2.2. Dealkylative cyanation

2.2.1. General Procedure: Dealkylative Cyanation of Sulfoxides

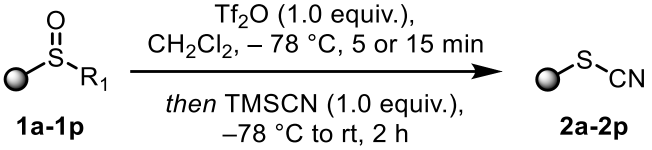

1a-1p **2a-2p**

A solution of sulfoxide (1.0 equiv.) in CH_2Cl_2 (0.1 M) was cooled to –78 °C. Trifluoromethanesulfonic anhydride (Tf_2O, 1.0 equiv.) was added and the mixture was stirred for 5 or 15 minutes at this temperature (5 minutes for substrates: **1a**-**1e**, **1h**-**1j**, **1l**-**1p** and 15 minutes for substrates: **1f**, **1g**, **1k**). After this time, trimethylsilyl cyanide (TMSCN, 1.0 equiv.) was added and the resulting mixture was allowed to warm to ambient temperature (23 °C) over the course of 2 h. The reaction was diluted to two times its volume with CH_2Cl_2 and solid sodium bicarbonate ($NaHCO_3$, 3.0 equiv.) was added. The suspension was stirred for 5 minutes, dried over anhydrous magnesium sulfate, filtered and the solvent removed under reduced pressure. The crude residue was purified by flash column chromatography (SiO_2, heptane/ethyl acetate) affording the products **2**.

1-methyl-4-thiocyanatobenzene (2a)

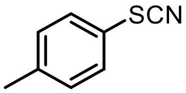

a) Following the general procedure using 1-methyl-4-(methylsulfinyl)benzene **1a** (72.0 mg, 0.47 mmol, 1.0 equiv.), trifluoromethanesulfonic anhydride (79 µL, 0.47 mmol, 1.0 equiv.), trimethylsilyl cyanide (58 µL, 0.47 mmol, 1.0 equiv.) and CH_2Cl_2 (4.7 mL). The title compound was obtained (63.5 mg, 91%) as a colourless liquid.
b) Following the general procedure using 1-(cyclohexylsulfinyl)-4-methylbenzene **1a'** (22.2 mg, 0.10 mmol, 1.0 equiv.), trifluoromethanesulfonic anhydride (17 µL, 0.10 mmol, 1.0 equiv.), trimethylsilyl cyanide (12.5 µL, 0.10 mmol, 1.0 equiv.) and CH_2Cl_2 (1.0 mL). The title compound was obtained (4.3 mg, 29%) as a colourless liquid.

Spectroscopic data was consistent with the literature.[16]

^{1}H NMR (400 MHz, CDCl$_3$): δ 7.43 (d, *J* = 8.2 Hz, 2H), 7.24 (d, *J* = 8.0 Hz, 2H), 2.38 (s, 3H).

^{13}C NMR (101 MHz, CDCl$_3$): δ 140.4, 131.1 (2C), 130.9 (2C), 120.7, 111.2, 21.3.

1-methyl-2-thiocyanatobenzene (2b)

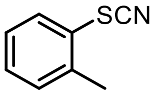

Following the general procedure using 1-methyl-2-(methylsulfinyl)benzene **1b** (15.4 mg, 0.10 mmol, 1.0 equiv.), trifluoromethanesulfonic anhydride (17 µL, 0.10 mmol, 1.0 equiv.), trimethylsilyl cyanide (12.5 µL, 0.10 mmol, 1.0 equiv.) and CH_2Cl_2 (1.0 mL). The title compound was obtained (10.0 mg, 67%) as a yellow oil. Spectroscopic data was consistent with the literature.[16]

^{1}H NMR (600 MHz, CDCl$_3$): δ 7.64 – 7.62 (m, 1H), 7.34 (td, *J* = 7.5, 1.2 Hz, 1H), 7.31 – 7.26 (m, 2H), 2.48 (s, 3H).

^{13}C NMR (151 MHz, CDCl$_3$): δ 139.5, 132.1, 131.6, 130.4, 127.9, 123.8, 110.6, 20.6.

1,3,5-trimethyl-2-thiocyanatobenzene (2c)

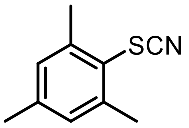

Following the general procedure using 1,3,5-trimethyl-2-(methylsulfinyl)benzene **1c** (18.2 mg, 0.10 mmol, 1.0 equiv.), trifluoromethanesulfonic anhydride (17 µL, 0.10 mmol, 1.0 equiv.), trimethylsilyl cyanide (12.5 µL, 0.10 mmol, 1.0 equiv.) and CH_2Cl_2 (1.0 mL). The title compound was obtained (17.6 mg, 99%) as a colourless liquid. Spectroscopic data was consistent with the literature.[17]

^{1}H NMR (600 MHz, $CDCl_3$): δ 7.01 (s, 2H), 2.55 (s, 6H), 2.30 (s, 3H).

^{13}C NMR (151 MHz, $CDCl_3$): δ 142.9 (2C), 141.6, 130.2 (2C), 119.2, 111.0, 22.0 (2C), 21.2.

1-bromo-4-thiocyanatobenzene (2d)

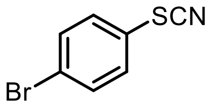

Following the general procedure using 1-bromo-4-(methylsulfinyl)benzene **2c** (21.9 mg, 0.10 mmol, 1.0 equiv.), trifluoromethanesulfonic anhydride (17 µL, 0.10 mmol, 1.0 equiv.), trimethylsilyl cyanide (12.5 µL, 0.10 mmol, 1.0 equiv.) and CH_2Cl_2 (1.0 mL). The title compound was obtained (13.8 mg, 65%) as colourless crystals. Spectroscopic data was consistent with the literature.[18]

^{1}H NMR (500 MHz, $CDCl_3$): δ 7.60 – 7.55 (m, 2H), 7.43 – 7.38 (m, 2H).

^{13}C NMR (126 MHz, $CDCl_3$): δ 133.6 (2C), 131.7 (2C), 124.3, 123.6, 110.0.

1-chloro-2-thiocyanatobenzene (2e)

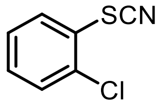

Following the general procedure using 1-chloro-2-(methylsulfinyl)benzene **1e** (17.5 mg, 0.10 mmol, 1.0 equiv.), trifluoromethanesulfonic anhydride (17 µL, 0.10 mmol, 1.0 equiv.), trimethylsilyl cyanide (12.5 µL, 0.10 mmol, 1.0 equiv.) and CH_2Cl_2 (1.0 mL). The title compound was obtained (14.9 mg, 88%) as colourless crystals. Spectroscopic data was consistent with the literature.[19]

^{1}H NMR (600 MHz, CDCl$_3$): δ 7.71 (dd, *J* = 7.8, 1.5 Hz, 1H), 7.46 (dd, *J* = 7.8, 1.3 Hz, 1H), 7.41 – 7.31 (m, 2H).

^{13}C NMR (151 MHz, CDCl$_3$): δ 132.9, 130.5, 130.3, 129.9, 128.6, 125.0, 109.4.

1,3-dichloro-2-thiocyanatobenzene (2f)

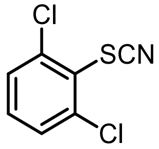

Following the general procedure using 1,3-dichloro-2-(methylsulfinyl)benzene **1f** (20.9 mg, 0.10 mmol, 1.0 equiv.), trifluoromethanesulfonic anhydride (17 µL, 0.10 mmol, 1.0 equiv.), trimethylsilyl cyanide (12.5 µL, 0.10 mmol, 1.0 equiv.) and CH_2Cl_2 (1.0 mL). The title compound was obtained (19.0 mg, 93%) as colourless crystals.

^{1}H NMR (600 MHz, CDCl$_3$): δ 7.50 (d, *J* = 8.1 Hz, 2H), 7.42 – 7.37 (m, 1H).

^{13}C NMR (151 MHz, CDCl$_3$): δ 140.7 (2C), 132.9, 129.6 (2C), 122.7, 108.3.

IR (neat) ν_{max}: 3070, 2161, 1557, 1422, 1401, 1190, 783, 734 cm^{-1}.

This compound was not detected using ESI ionization. **LRMS (EI$^+$):** mass calculated for $[M]^+$ ($C_7H_3Cl_2NS$) *m/z* 202.9, found *m/z* 202.9. Fragmentation pattern: 206.9 (13), 204.9 (68), 202.9 (100), 170.0 (29), 168.0 (77), 142.0 (19), 133.0 (24).

M$_p$: 61–63 °C.

1,3-difluoro-2-thiocyanatobenzene (2g)

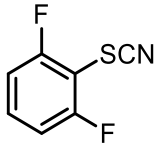

Following the general procedure using 1,3-difluoro-2-(methylsulfinyl)benzene **1g** (17.6 mg, 0.10 mmol, 1.0 equiv.), trifluoromethanesulfonic anhydride (17 µL, 0.10 mmol, 1.0 equiv.), trimethylsilyl cyanide (12.5 µL, 0.10 mmol, 1.0 equiv.) and CH_2Cl_2 (1.0 mL). The title compound was obtained (13.1 mg, 76%) as a colourless crystals.

^{1}H NMR (600 MHz, $CDCl_3$): δ 7.52 (tt, *J* = 8.5, 6.3 Hz, 1H), 7.12 – 7.05 (m, 2H).

^{13}C NMR (151 MHz, $CDCl_3$): δ 162.3 (C-F, $^1J_{C-F}$ = 255.1, $^3J_{C-F}$ = 3.0 Hz, 2C), 134.0 (C-F, $^3J_{C-F}$ = 10.1 Hz), 112.8 (C-F, $^2J_{C-F}$ = 21.9, $^4J_{C-F}$ = 3.7 Hz, 2C), 108.3, 100.5 (C-F, $^2J_{C-F}$ = 21.6 Hz).

^{19}F NMR (565 MHz, $CDCl_3$): δ -102.34 (t, *J* = 6.7 Hz).

IR (neat) ν_{max}: 2953, 2349, 2323, 2165, 1606, 1469, 1288, 1241, 999, 787, 712, 671, 664, 606 cm^{-1}.

This compound was not detected using ESI ionization. **LRMS (EI$^+$):** mass calculated for $[M]^+$ ($C_7H_3F_2NS$) *m/z* 171.0, found *m/z* 171.0. Fragmentation pattern: 173.0 (5), 172.0 (9), 171.0 (100), 151.0 (27), 127.1 (27), 101.1 (25), 63.1 (17).

M_p: 41–43 °C.

2-thiocyanatonaphthalene (2h)

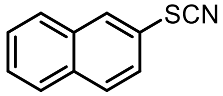

Following the general procedure using 2-(methylsulfinyl)naphthalene **2h** (19.0 mg, 0.10 mmol, 1.0 equiv.), trifluoromethanesulfonic anhydride (17 µL, 0.10 mmol, 1.0 equiv.), trimethylsilyl cyanide (12.5 µL, 0.10 mmol, 1.0 equiv.) and CH_2Cl_2 (1.0 mL). The title compound was obtained (16.4 mg, 89%) as colourless crystals. Spectroscopic data was consistent with the literature.[20]

^{1}H NMR (600 MHz, $CDCl_3$): δ 8.03 (d, *J* = 1.3 Hz, 1H), 7.91 (d, *J* = 8.7 Hz, 1H), 7.86 (dt, *J* = 6.9, 3.6 Hz, 1H), 7.85 – 7.81 (m, 1H), 7.59 – 7.54 (m, 3H).

^{13}C NMR (151 MHz, $CDCl_3$): δ 133.8, 133.2, 130.4, 130.0, 128.1, 127.8, 127.8, 127.7, 126.4, 121.5, 110.8.

1-methoxy-2-thiocyanatobenzene (2i)

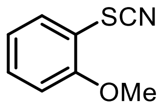

a) Following the general procedure using 1-methoxy-2-(methylsulfinyl)benzene **1i** (17.0 mg, 0.10 mmol, 1.0 equiv.), trifluoromethanesulfonic anhydride (17 µL, 0.10 mmol, 1.0 equiv.), trimethylsilyl cyanide (12.5 µL, 0.10 mmol, 1.0 equiv.) and CH_2Cl_2 (1.0 mL). The title compound was obtained (16.5 mg, quantitative) as a yellow oil.
b) Following the general procedure using 1-(cyclohexylsulfinyl)-2-methoxybenzene **1i'** (23.8 mg, 0.10 mmol, 1.0 equiv.), trifluoromethanesulfonic anhydride (17 µL, 0.10 mmol, 1.0 equiv.), trimethylsilyl cyanide (12.5 µL, 0.10 mmol, 1.0 equiv.) and CH_2Cl_2 (1.0 mL). The title compound was obtained (8.0 mg, 48%) as a yellow oil.

Spectroscopic data was consistent with the literature.[16]

^{1}H NMR (600 MHz, $CDCl_3$): δ 7.57 (dd, *J* = 7.8, 1.4 Hz, 1H), 7.36 (td, *J* = 8.3, 1.5 Hz, 1H), 7.06 – 7.02 (m, 1H), 6.94 (d, *J* = 8.2 Hz, 1H), 3.92 (s, 3H).

^{13}C NMR (151 MHz, $CDCl_3$): δ 156.6, 130.7, 130.1, 122.2, 113.3, 111.6, 110.6, 56.3.

1,3-dimethoxy-2-thiocyanatobenzene (2j)

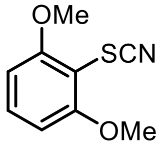

a) Following the general procedure using 1,3-dimethoxy-2-(methylsulfinyl)benzene **1j** (100 mg, 0.50 mmol, 1.0 equiv.), trifluoromethanesulfonic anhydride (84 µL, 0.50 mmol, 1.0 equiv.), trimethylsilyl cyanide (63 µL, 0.50 mmol, 1.0 equiv.) and CH_2Cl_2 (5.0 mL). The title compound was obtained (78.3 mg, 80%) as colourless crystals.
b) Scale up experiment: Following the general procedure using 1,3-dimethoxy-2-(methylsulfinyl)benzene **1j** (1.202 g, 6.00 mmol, 1.0 equiv.), commercial trifluoromethanesulfonic anhydride (1.0 mL, 6.00 mmol, 1.0 equiv.), trimethylsilyl cyanide (0.75 mL, 6.00 mmol, 1.0 equiv.) and CH_2Cl_2 (60.0 mL). After the solvent was removed under reduced pressure, the remaining solid was titurated with warm methyl tert-butyl ether (4x50 mL) and decanted leaving behind an oily residue. The combined methyl tert-butyl ether phases were concentrated *in vacuo* to give title compound (1.027 g, 88%) as colourless crystals.

^{1}H NMR (400 MHz, $CDCl_3$): δ 7.37 (t, *J* = 8.4 Hz, 1H), 6.60 (d, *J* = 8.4 Hz, 2H), 3.92 (s, 6H).

^{13}C NMR (101 MHz, $CDCl_3$): δ 160.1 (2C), 132.8, 111.1, 104.5 (2C), 98.9, 56.5 (2C).

IR (neat) ν_{max}: 2152, 1576, 1474, 1431, 1256, 1100, 1019, 774, 664, 600 cm^{-1}.

HRMS (ESI$^+$): exact mass calculated for $[M+Na]^+$ ($C_9H_9NO_2SNa$) *m/z* 218.0246, found *m/z* 218.0243.

M_p: 103–105 °C.

1-nitro-3-thiocyanatobenzene (2k)

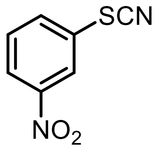

Following the general procedure using 1-(methylsulfinyl)-3-nitrobenzene **1k** (18.5 mg, 0.10 mmol, 1.0 equiv.), trifluoromethanesulfonic anhydride (17 µL, 0.10 mmol, 1.0 equiv.), trimethylsilyl cyanide (12.5 µL, 0.10 mmol, 1.0 equiv.) and CH_2Cl_2 (1.0 mL). The title compound was obtained (10.1 mg, 56%) as colourless crystals. Spectroscopic data was consistent with the literature.[21]

^{1}H NMR (600 MHz, CDCl$_3$): δ 8.39 (t, *J* = 2.1 Hz, 1H), 8.28 (ddd, *J* = 8.3, 2.2, 0.9 Hz, 1H), 7.88 (ddd, *J* = 7.9, 2.0, 1.0 Hz, 1H), 7.68 (t, *J* = 8.1 Hz, 1H).

^{13}C NMR (151 MHz, CDCl$_3$): δ 149.1, 135.0, 131.4, 127.5, 124.5 (2C), 108.7.

methyl 3-methoxy-4-thiocyanato-2-naphthoate (2l)

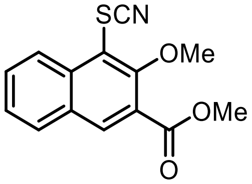

Following the general procedure using methyl 3-methoxy-4-(methylsulfinyl)-2-naphthoate **1l** (55.7 mg, 0.20 mmol, 1.0 equiv.), trifluoromethanesulfonic anhydride (34 µL, 0.20 mmol, 1.0 equiv.), trimethylsilyl cyanide (25 µL, 0.20 mmol, 1.0 equiv.) and CH_2Cl_2 (2.0 mL). The title compound was obtained (28.4 mg, 52%) as colourless crystals.

^{1}H NMR (400 MHz, CDCl$_3$): δ 8.58 (s, 1H), 8.38 (d, *J* = 8.7 Hz, 1H), 7.96 (d, *J* = 8.2 Hz, 1H), 7.83 – 7.77 (m, 1H), 7.64 – 7.57 (m, 1H), 4.11 (s, 3H), 4.01 (s, 3H).

^{13}C NMR (101 MHz, CDCl$_3$): δ 165.5, 158.9, 137.6, 135.7, 130.9, 130.3, 130.1, 127.0, 125.0, 124.8, 114.4, 110.7, 63.5, 52.9.

IR (neat) ν_{max}: 2947, 2158, 1728, 1448, 1336, 1275, 1236, 1149, 1078, 1002, 756 cm^{-1}.
HRMS (ESI$^+$): exact mass calculated for [M+Na]$^+$ ($C_{14}H_{11}NO_3SNa$) *m/z* 296.0352, found *m/z* 296.0354.
M$_p$: 96–98 °C.

1-thiocyanatooctane (2m)

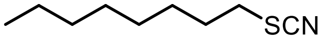

Following the general procedure using 1-(methylsulfinyl)octane **1m** (17.6 mg, 0.10 mmol, 1.0 equiv.), trifluoromethanesulfonic anhydride (17 µL, 0.10 mmol, 1.0 equiv.), trimethylsilyl cyanide

(12.5 µL, 0.10 mmol, 1.0 equiv.) and CH_2Cl_2 (1.0 mL). The title compound was obtained (9.7 mg, 57%) as a colourless liquid. Spectroscopic data was consistent with the literature.[21]

^{1}H NMR (600 MHz, $CDCl_3$): δ 2.95 (t, *J* = 7.3 Hz, 2H), 1.86 – 1.78 (m, 2H), 1.47 – 1.39 (m, 2H), 1.36 – 1.22 (m, 8H), 0.92 – 0.81 (m, 3H).

^{13}C NMR (151 MHz, $CDCl_3$): δ 112.6, 34.2, 31.8, 30.0, 29.2, 29.0, 28.1, 22.7, 14.2.

3,5-dimethyl-4-thiocyanatophenyl methylcarbamate (2n)

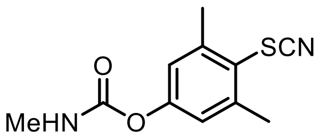

Following the general procedure using 3,5-dimethyl-4-(methylsulfinyl)phenyl methylcarbamate **1n** (Methiocarb sulfoxide, 33.8 mg, 0.14 mmol, 1.0 equiv.), trifluoromethanesulfonic anhydride (24 µL, 0.14 mmol, 1.0 equiv.), trimethylsilyl cyanide (17.5 µL, 0.14 mmol, 1.0 equiv.) and CH_2Cl_2 (1.5 mL). The title compound was obtained (24.0 mg, 73%) as beige crystals along with the deprotected 3,5-dimethyl-4-thiocyanatophenol (8.4 mg, 25%) as colourless crystals. The title compound appears in ^{1}H-NMR as a mixture of rotamers denoted as R1 (major) and R2 (minor) in a ratio of 7.6:1.

^{1}H NMR (600 MHz, $CDCl_3$): δ 7.02 (s, 2H, C_{Ar}–H_{R2}), 6.99 (s, 2H, C_{Ar}–H_{R1}), 5.04 (s, 1H, NH_{R1}), 4.77 (s, 1H, NH_{R2}), 2.95 (d, *J* = 4.7 Hz, 3H, NMe_{R2}), 2.89 (d, *J* = 4.9 Hz, 3H, NMe_{R1}), 2.59 (s, 6H, $ArMe_{R2}$), 2.58 (s, 6H, $ArMe_{R1}$).

^{13}C NMR (151 MHz, $CDCl_3$): δ 154.5, 152.9, 144.5 (2C), 122.2 (2C), 118.7, 110.3, 27.7, 22.1 (2C).

IR (neat) ν_{max}: 3346, 2946, 2155, 1727, 1525, 1459, 1300, 1239, 1158 cm^{-1}.

HRMS (ESI$^+$): exact mass calculated for $[M+Na]^+$ ($C_{11}H_{12}N_2O_2SNa$) *m/z* 259.0512, found *m/z* 259.0512.

M_p: 140–142 °C.

(2-thiocyanatoethyl)benzene (2o)

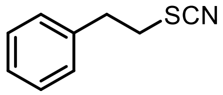

a) Following the general procedure using (2-(methylsulfinyl)ethyl)benzene **1o** (33.7 mg, 0.20 mmol, 1.0 equiv.), trifluoromethanesulfonic anhydride (34 µL, 0.20 mmol, 1.0 equiv.), trimethylsilyl cyanide (25 µL, 0.20 mmol, 1.0 equiv.) and CH_2Cl_2 (1.0 mL). Analysis of the crude ^{1}H NMR showed quantitative formation of phenethyl trifluoromethanesulfonate (homobenzyl triflate) using mesitylene as internal standard, (2-thiocyanatoethyl)benzene **2o** was not detected.

b) Following the general procedure using (2-(benzylsulfinyl)ethyl)benzene **1o'** (23.8 mg, 0.20 mmol, 1.0 equiv.), trifluoromethanesulfonic anhydride (34 µL, 0.20 mmol, 1.0 equiv.), trimethylsilyl cyanide (25 µL, 0.20 mmol, 1.0 equiv.) and CH_2Cl_2 (1.0 mL). Analysis of the crude ^{1}H NMR showed the formation of (2-thiocyanatoethyl)benzene **2o** in 51% NMR yield using mesitylene as internal standard.

c) Following the general procedure using (sulfinylbis(ethane-2,1-diyl))dibenzene **1o''** (51.7 mg, 0.20 mmol, 1.0 equiv.), trifluoromethanesulfonic anhydride (34 µL, 0.20 mmol, 1.0 equiv.), trimethylsilyl cyanide (25 µL, 0.20 mmol, 1.0 equiv.) and CH_2Cl_2 (2.0 mL). The title compound was obtained (28.6 mg, 88%) as a colourless oil.

Spectroscopic data was consistent with the literature.[22]

^{1}H NMR (600 MHz, $CDCl_3$): δ 7.39 – 7.32 (m, 2H), 7.32 – 7.27 (m, 1H), 7.26 – 7.19 (m, 2H), 3.22 – 3.16 (m, 2H), 3.15 – 3.08 (m, 2H).

^{13}C NMR (151 MHz, $CDCl_3$): δ 137.8, 129.0 (2C), 128.8 (2C), 127.4, 112.2, 36.2, 35.3.

3-(2-thiocyanatophenyl)propyl trifluoromethanesulfonate (2p)

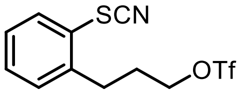

Following the general procedure using thiochromane 1-oxide **1p** (16.6 mg, 0.10 mmol, 1.0 equiv.), trifluoromethanesulfonic anhydride (17 µL, 0.10 mmol, 1.0 equiv.), trimethylsilyl cyanide (12.5 µL, 0.10 mmol, 1.0 equiv.) and CH_2Cl_2 (1.0 mL). The title compound was obtained (31.8 mg, 98%) as a colourless oil.

^{1}H NMR (600 MHz, CDCl$_3$): δ 7.71 (d, *J* = 7.8 Hz, 1H), 7.43 (t, *J* = 7.5 Hz, 1H), 7.39 – 7.29 (m, 2H), 4.60 (t, *J* = 6.0 Hz, 2H), 3.02 – 2.94 (m, 2H), 2.20 (tt, *J* = 12.4, 6.1 Hz, 2H).

^{13}C NMR (151 MHz, CDCl$_3$): δ 141.2, 133.5, 131.1, 130.9, 129.0, 123.6, 118.8 (C-F, $^1J_{C-F}$ = 319.5 Hz), 110.5, 76.1, 29.9, 29.8.

^{19}F NMR (565 MHz, CDCl$_3$): δ -74.61.

IR (neat) ν_{max}: 2938, 2865, 2158, 1467, 1411, 1245, 1209, 1143, 1116, 1030, 930, 756 cm^{-1}.

HRMS (ESI$^+$): exact mass calculated for $[M+H]^+$ ($C_{11}H_{11}F_3NO_3S_2$) *m/z* 326.0127, found *m/z* 326.1030.

2.3. One-pot transformations

hex-1-yn-1-yl(naphthalen-2-yl)sulfane (3)

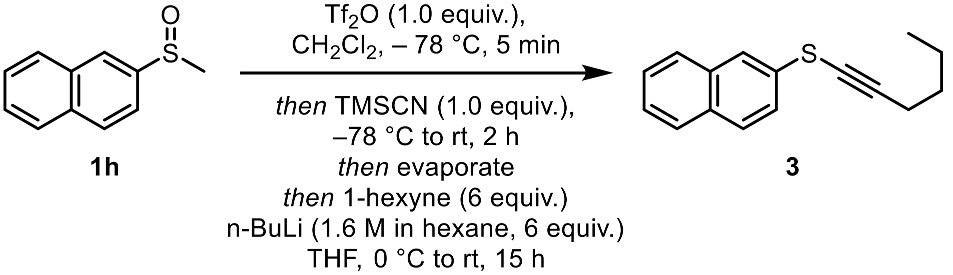

A solution of 2-(methylsulfinyl)naphthalene **1h** (28.5 mg, 0.15 mmol, 1.0 equiv.) in CH_2Cl_2 (1.5 mL, 0.1 M) was cooled –78 °C. Trifluoromethanesulfonic anhydride (25 µL, 0.15 mmol, 1.0 equiv.) was added and the mixture was stirred for 5 minutes at this temperature. After this time, trimethylsilyl cyanide (19 µL, 0.15 mmol, 1.0 equiv.) was added and the resulting mixture was

allowed to warm to ambient temperature (23 °C) over the course of 2 h. In a separate flame-dried Schlenk tube, a solution of 1-hexyne (103 µL, 0.90 mmol, 6.0 equiv.) in THF (4.5 mL) was cooled to –78 °C and n-BuLi (1.6 M in hexane, 0.56 mL, 0.90 mmol, 6.0 equiv.) was added dropwise. This solution was stirred for 30 minutes at –78 °C then warmed to 0 °C. The volatiles of the reaction mixture containing the crude thiocyanate were removed *in vacuo* and the flask containing the crude material was placed under argon in an ice bath. The solution of lithium alkynylide in THF was transferred using a syringe and the reaction was allowed to warm to ambient temperature (23 °C) and stirred for 15 h. A saturated aqueous solution of ammonium chloride (4 mL) was added and the reaction mixture was extracted with ethyl acetate (2 × 5 mL). The combined organic phases were dried over anhydrous magnesium sulfate, filtered and the solvent removed under reduced pressure. The crude residue was purified by flash column chromatography (SiO_2, heptane/ethyl acetate) affording the product (28.9 mg, 80%) as a yellowish oil.

^{1}H NMR (600 MHz, $CDCl_3$): δ 7.88 (d, *J* = 1.6 Hz, 1H), 7.80 (t, *J* = 7.8 Hz, 2H), 7.76 (d, *J* = 8.0 Hz, 1H), 7.51 – 7.42 (m, 3H), 2.52 (t, *J* = 7.1 Hz, 2H), 1.68 – 1.62 (m, 2H), 1.57 – 1.49 (m, 2H), 0.98 (t, *J* = 7.3 Hz, 3H).

^{13}C NMR (151 MHz, $CDCl_3$): δ 133.9, 132.0, 131.3, 128.8, 128.0, 127.2, 126.9, 125.9, 124.1, 124.0, 100.5, 64.8, 30.9, 22.2, 20.2, 13.8.

IR (neat) ν_{max}: 3053, 2928, 2865, 1588, 1501, 1457, 1134, 1067, 944, 849, 809, 742 cm^{-1}.

This compound was not detected using ESI ionization. **LRMS (EI$^+$):** mass calculated for $[M]^+$ ($C_{16}H_{16}S$) *m/z* 240.1, found *m/z* 240.1. Fragmentation pattern: 242.1 (5), 241.1 (18), 240.1 (100), 197.1 (44), 160.1 (36), 153.1 (52), 152.1 (36).

hex-1-yn-1-yl(naphthalen-2-yl)sulfane (4)

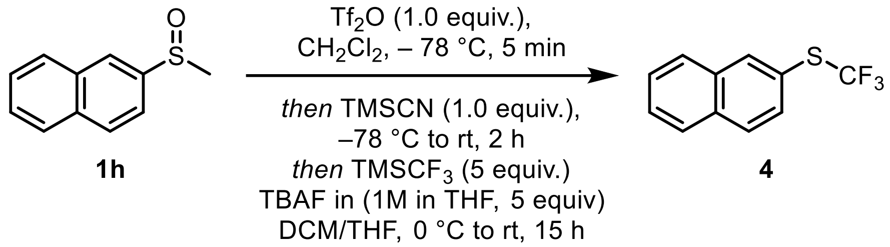

A solution of 2-(methylsulfinyl)naphthalene **1h** (28.5 mg, 0.15 mmol, 1.0 equiv.) in CH_2Cl_2 (1.5 mL, 0.1 M) was cooled –78 °C. Trifluoromethanesulfonic anhydride (25 µL, 0.15 mmol, 1.0 equiv.) was added and the mixture was stirred for 5 minutes at this temperature. After this time, trimethylsilyl cyanide (19 µL, 0.15 mmol, 1.0 equiv.) was added and the resulting mixture was allowed to warm to ambient temperature (23 °C) over the course of 2 h. The reaction was placed in an ice bath and (trifluoromethyl)trimethylsilane (111 µL, 0.75 mmol, 5.0 equiv.) was added followed by a dropwise addition of a solution of TBAF (1 M in THF, 0.75 mL, 0.75 mmol, 5.0 equiv.). The reaction was allowed to warm to ambient temperature (23 °C) and stirred for 15 h. A saturated aqueous solution of ammonium chloride (1 mL) was added and the reaction mixture was extracted with ethyl acetate (2 × 2 mL). The combined organic phases were dried over anhydrous magnesium sulfate, filtered and the solvent removed under reduced pressure. The crude residue was purified by flash column chromatography (SiO_2, heptane/ethyl acetate) affording the product (16.8 mg, 49%) as a colourless oil. Spectroscopic data was consistent with the literature.[23]

^{1}H NMR (600 MHz, $CDCl_3$): δ 8.21 (d, J = 1.0 Hz, 1H), 7.91 – 7.82 (m, 3H), 7.67 (dd, J = 8.6, 1.5 Hz, 1H), 7.62 – 7.54 (m, 2H).

^{13}C NMR (151 MHz, $CDCl_3$): δ 137.0, 133.9, 133.4, 131.8, 129.7 (CF, $^1J_{C\text{-}F}$ = 308.5 Hz), 129.2, 128.2, 127.9, 127.8, 127.0, 121.5 (CF, $^3J_{C\text{-}F}$ = 1.8 Hz).

^{19}F NMR (565 MHz, $CDCl_3$): δ -42.49.

naphthalene-2-sulfonyl cyanide (5)

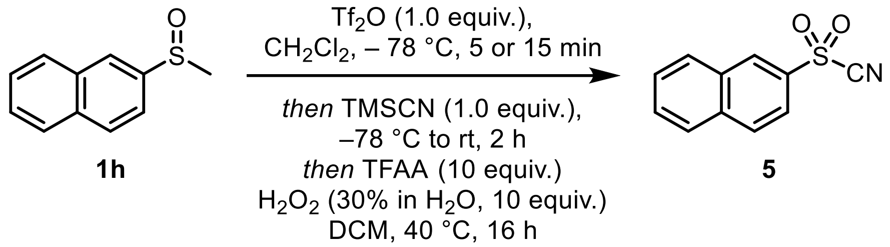

A solution of 2-(methylsulfinyl)naphthalene **1h** (47.6 mg, 0.25 mmol, 1.0 equiv.) in CH_2Cl_2 (2.5 mL, 0.1 M) was cooled –78 °C. Trifluoromethanesulfonic anhydride (44 µL, 0.25 mmol, 1.0 equiv.) was added and the mixture was stirred for 5 minutes at this temperature. After this time, trimethylsilyl cyanide (31 µL, 0.25 mmol, 1.0 equiv.) was added and the resulting mixture was allowed to warm to ambient temperature (23 °C) over the course of 2 h. In a separate round bottom flask, a solution of trifluoroacetic anhydride (TFAA, 0.35 mL, 2.5 mmol, 10 equiv.) in CH_2Cl_2 (2.5 mL) was cooled to 0 °C and treated dropwise with hydrogen peroxide (30 wt.% in H_2O, 0.25 mL, 2.5 mmol, 10 equiv.).[24] The mixture was stirred for 45 minutes at 0 °C then, the crude thiocyanate solution in DCM was transferred from the other flask using a syringe. The round bottom flask was closed with a glass lid and placed in a sand bath (40 °C). The reaction mixture was stirred at this temperature for 16 h, when water (5 mL) was added. The reaction was extracted with DCM (2 x 5 mL) and the combined organic phases were dried over anhydrous magnesium sulfate. Filtration and evaporation of the solvent removed under reduced pressure afforded a crude residue which was purified by flash column chromatography (SiO_2, heptane/ethyl acetate) affording the product (32.9 mg, 61%) as colourless crystals.

^{1}H NMR (400 MHz, $CDCl_3$): δ 8.69 (d, *J* = 1.5 Hz, 1H), 8.14 (d, *J* = 8.8 Hz, 1H), 8.09 (d, *J* = 8.2 Hz, 1H), 8.04 – 7.96 (m, 2H), 7.85 – 7.79 (m, 1H), 7.78 – 7.72 (m, 1H).

^{13}C NMR (101 MHz, $CDCl_3$): δ 136.8, 134.0, 132.5, 132.1, 131.5, 131.1, 130.2, 128.9, 128.5, 122.0, 114.3.

IR (neat) ν_{max}: 3065, 2927, 2183, 1377, 1176, 674 cm^{-1}.

This compound was not detected using ESI ionization. **LRMS (EI$^+$):** mass calculated for $[M]^+$ ($C_{11}H_7NO_sS$) *m/z* 217.0, found *m/z* 217.0. Fragmentation pattern: 218.0 (10), 217.0 (79), 153.1 (63), 128.1 (16), 127.1 (100), 126.1 (33).

M_p: 48–50 °C.

3. NMR Spectra

methyl 3-methoxy-4-(methylsulfinyl)-2-naphthoate (1l) – ^{1}H NMR (400 MHz)

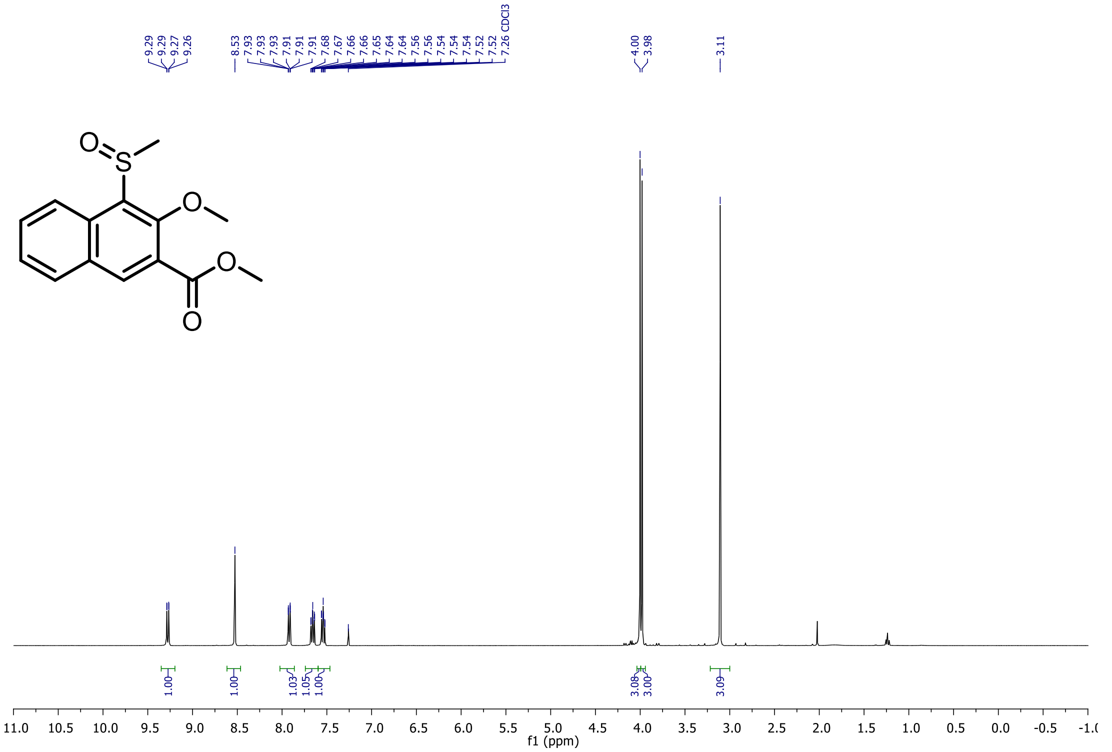

methyl 3-methoxy-4-(methylsulfinyl)-2-naphthoate (1l) – ^{13}C NMR (101 MHz)

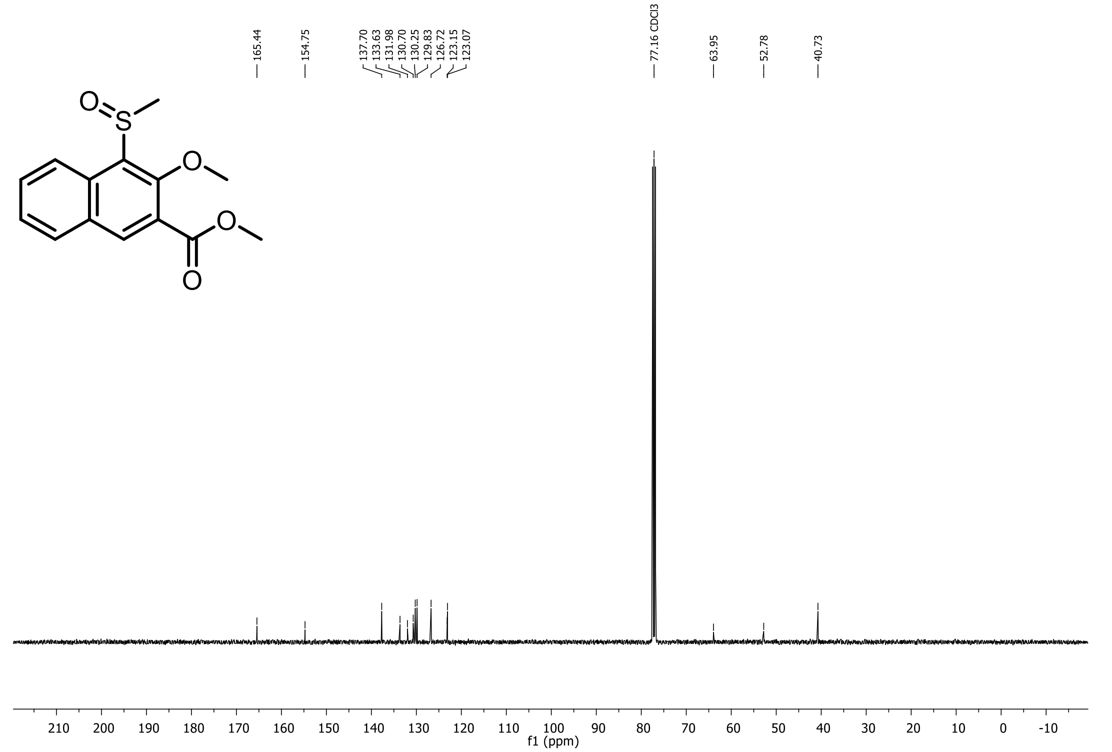

1-methyl-4-thiocyanatobenzene (2a) – ^{1}H NMR (400 MHz)

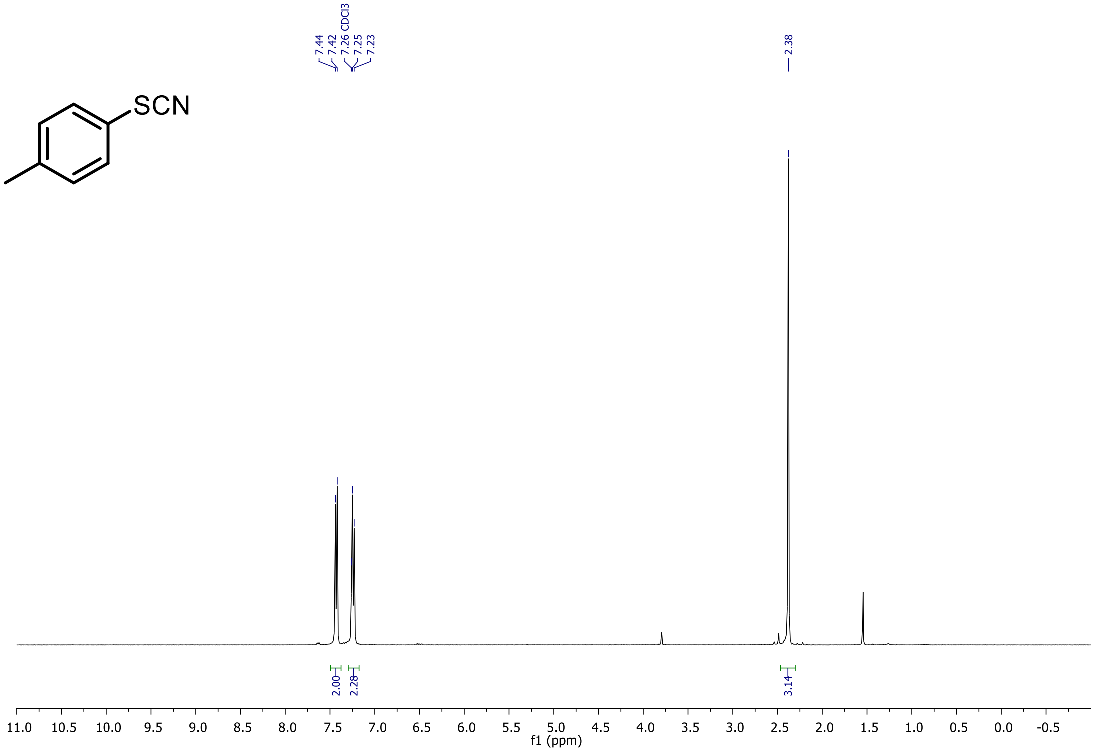

1-methyl-4-thiocyanatobenzene (2a) – ^{13}C NMR (101 MHz)

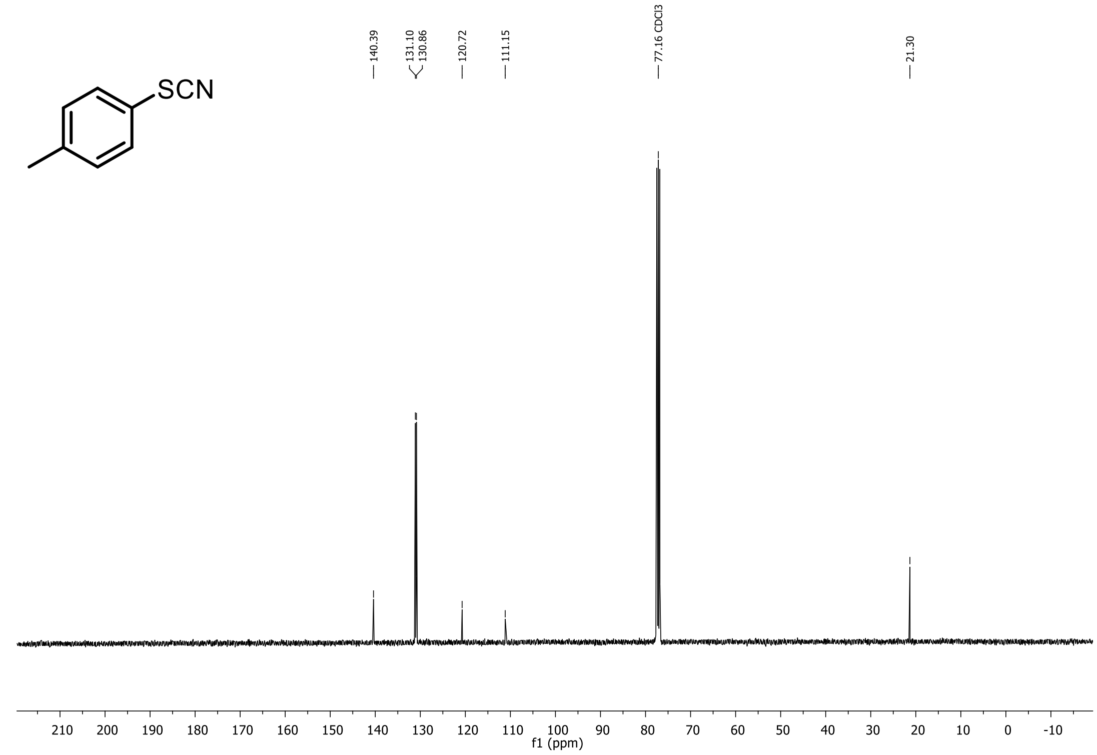

1-methyl-2-thiocyanatobenzene (2b) – ^{1}H NMR (600 MHz)

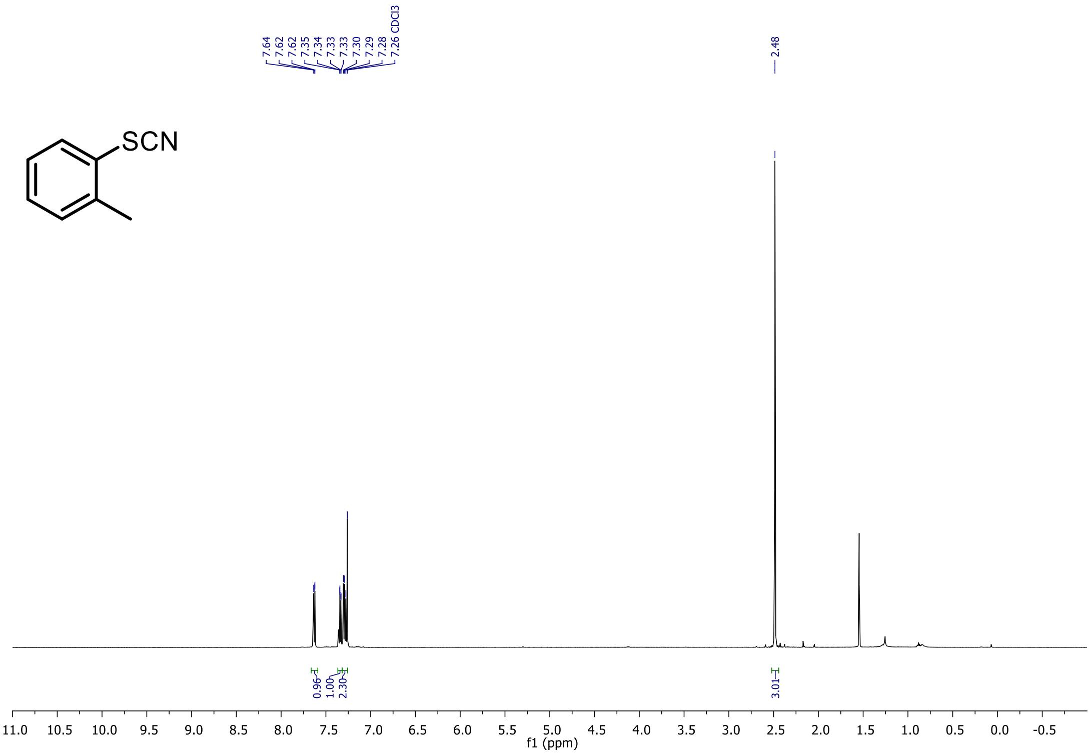

1-methyl-2-thiocyanatobenzene (2b) – ^{13}C NMR (151 MHz)

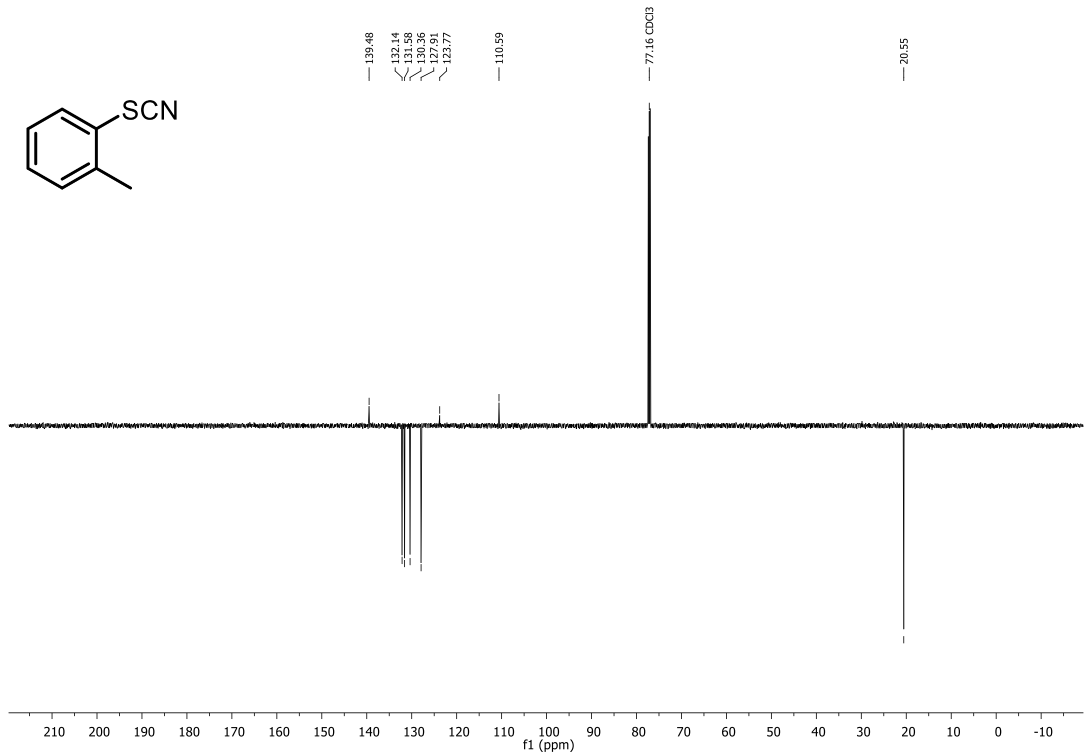

1,3,5-trimethyl-2-thiocyanatobenzene (2c) – ^{1}H NMR (600 MHz)

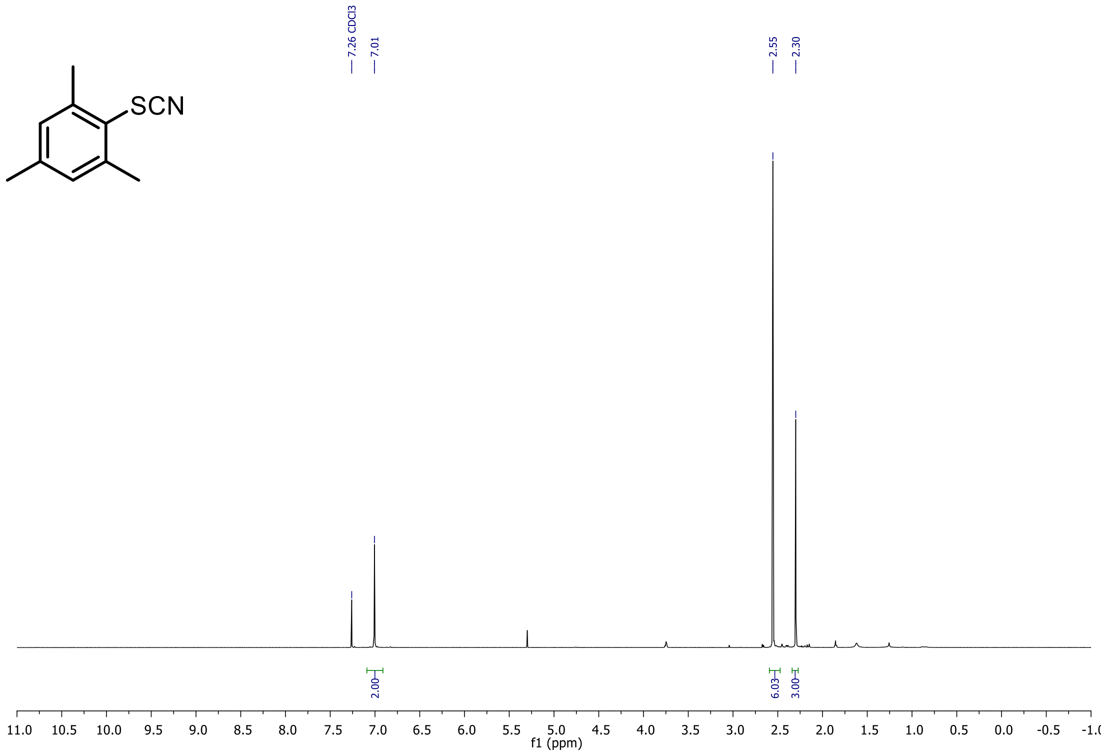

1,3,5-trimethyl-2-thiocyanatobenzene (2c) – ^{13}C NMR (151 MHz)

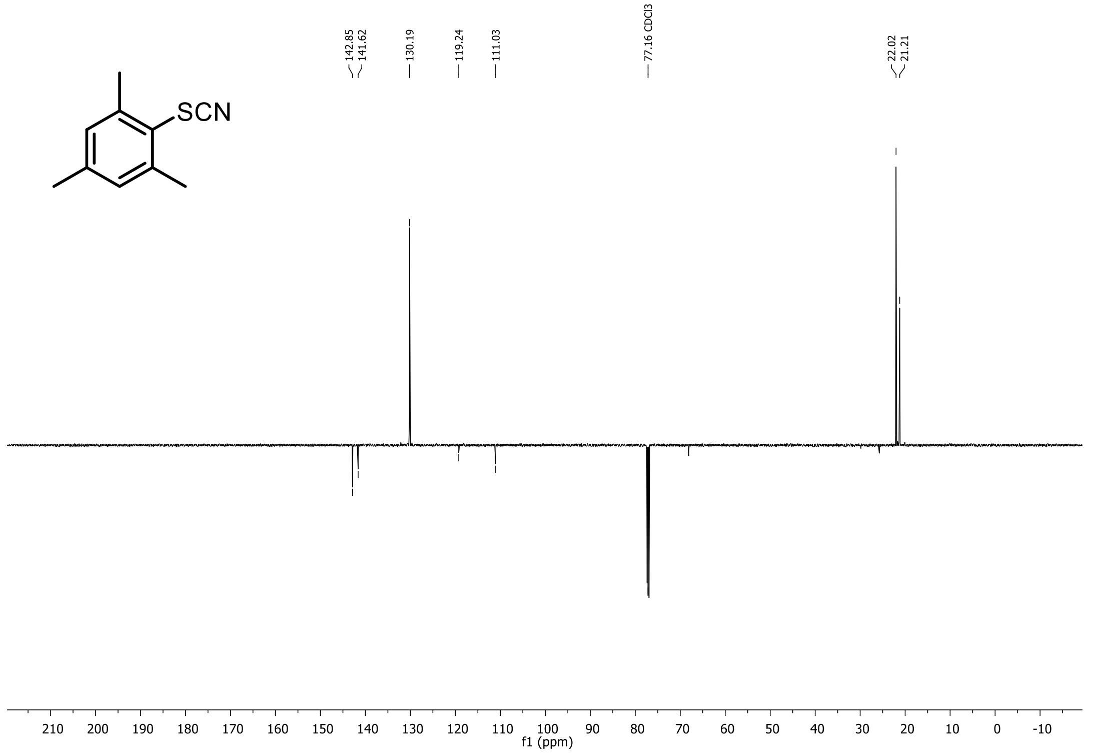

1-bromo-4-thiocyanatobenzene (2d) – ^{1}H NMR (500 MHz)

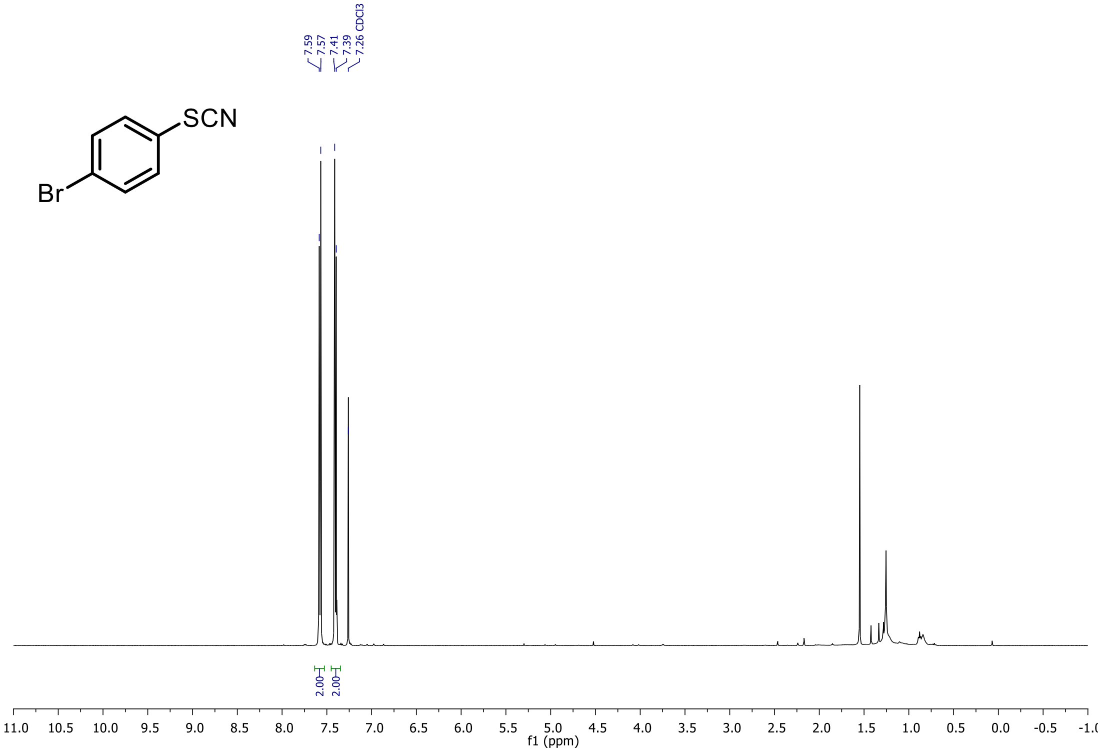

1-bromo-4-thiocyanatobenzene (2d) – ^{13}C NMR (126 MHz)

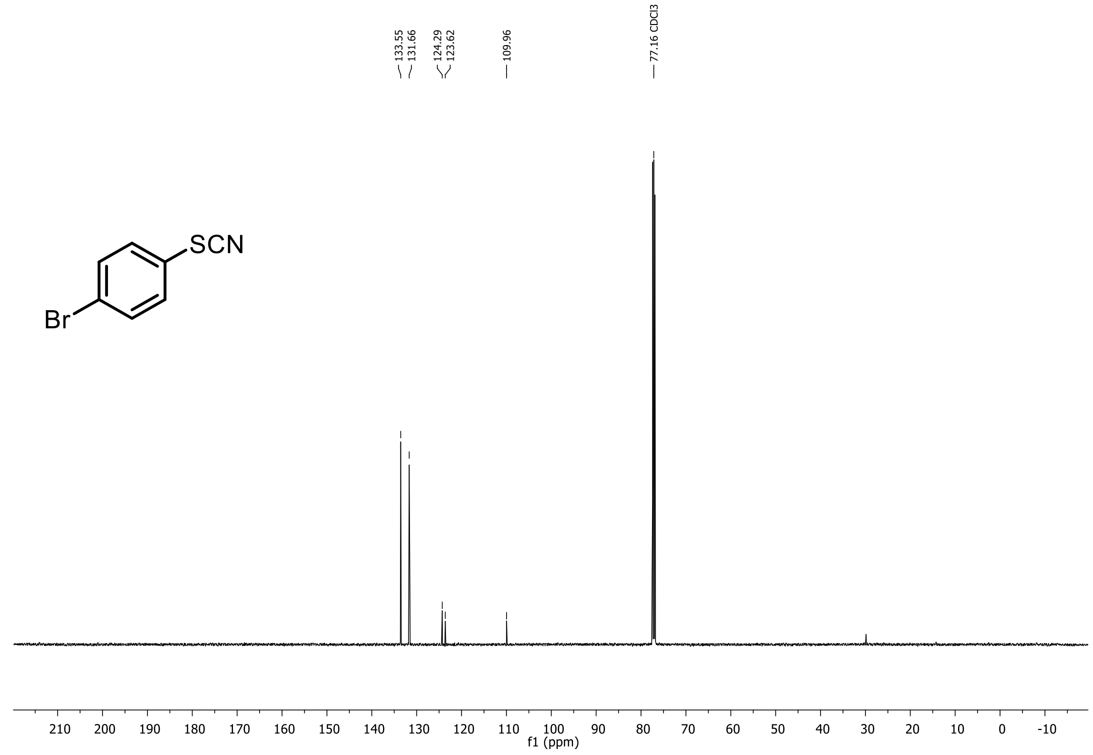

1-chloro-2-thiocyanatobenzene (2e) – ^{1}H NMR (600 MHz)

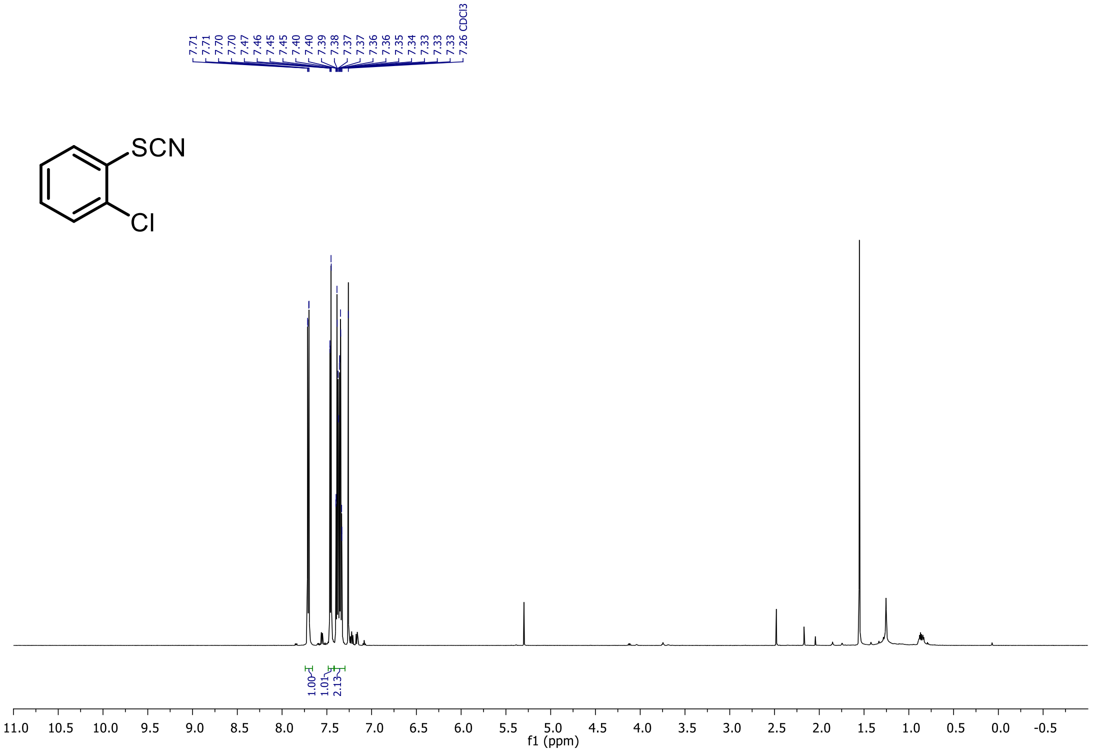

1-chloro-2-thiocyanatobenzene (2e) – ^{13}C NMR (151 MHz)

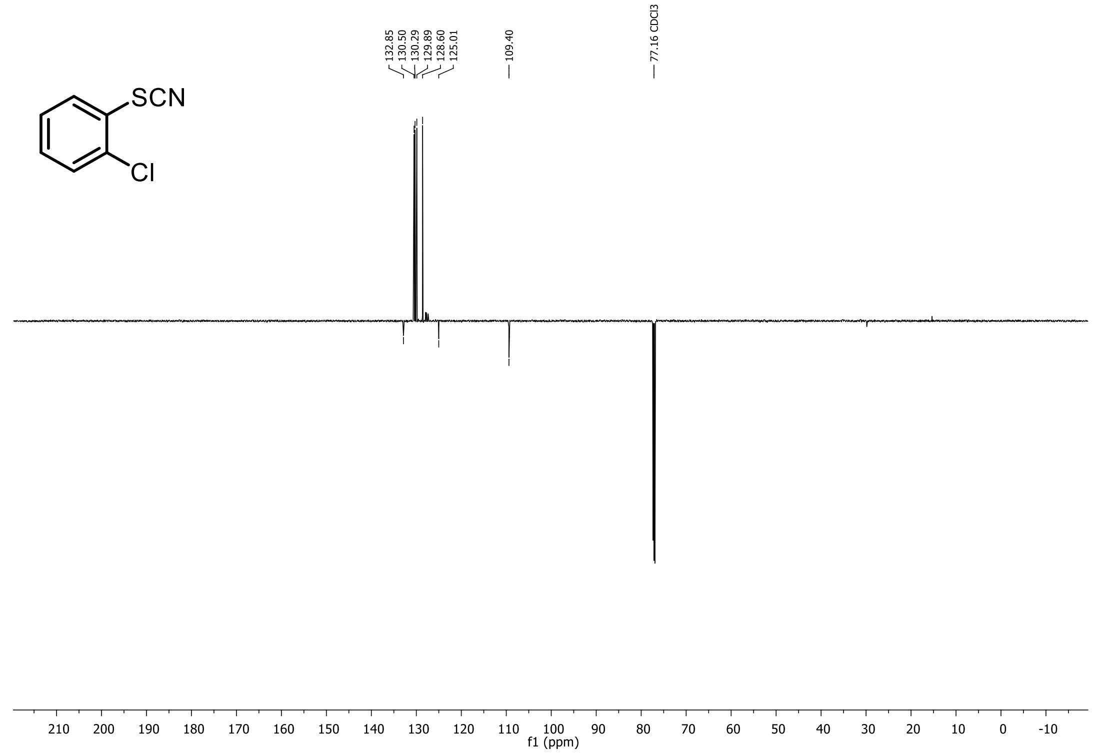

1,3-dichloro-2-thiocyanatobenzene (2f) – ^{1}H NMR (600 MHz)

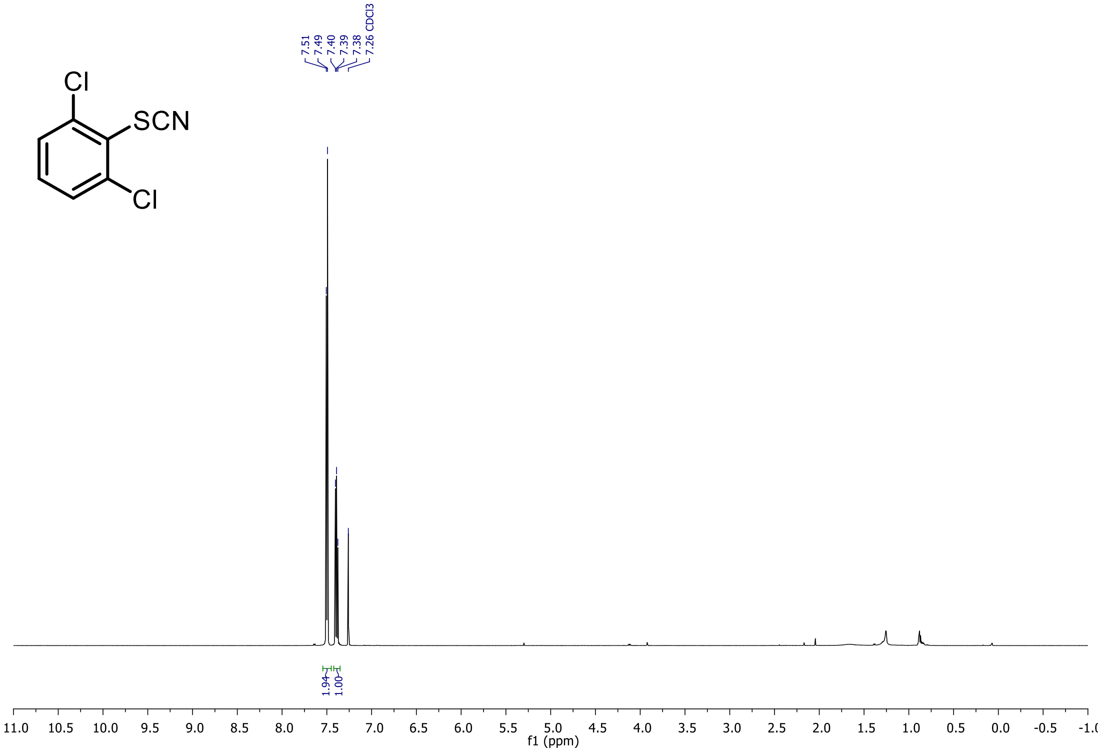

1,3-dichloro-2-thiocyanatobenzene (2f) – ^{13}C NMR (151 MHz)

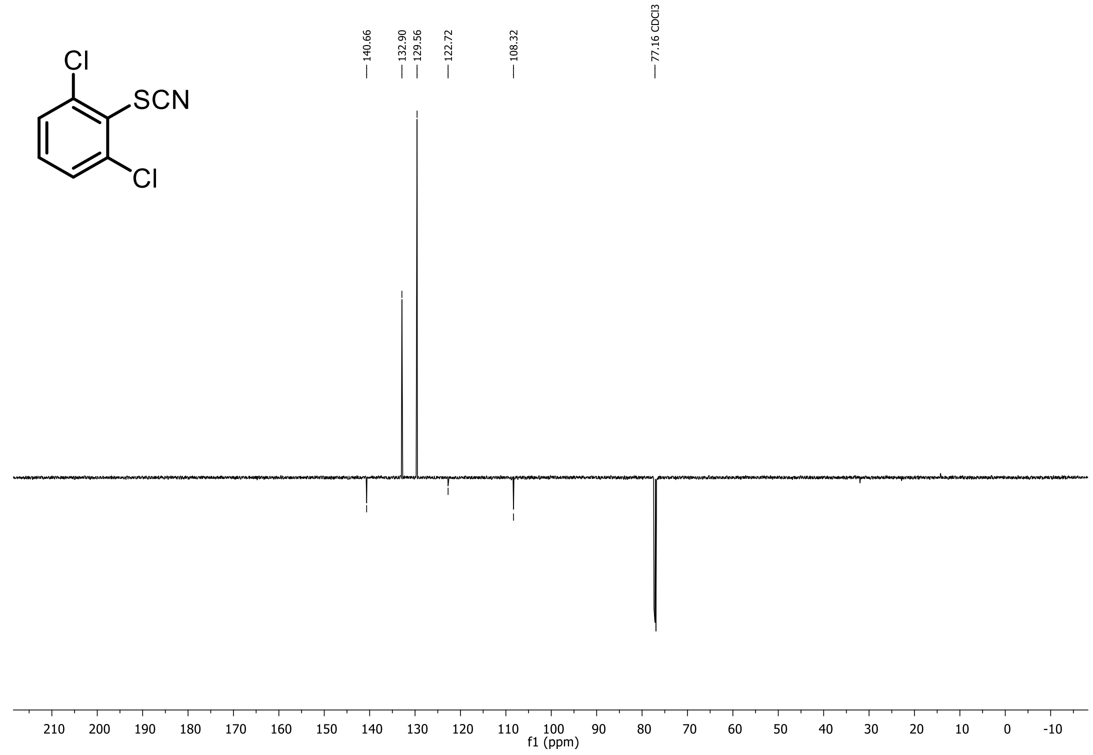

1,3-difluoro-2-thiocyanatobenzene (2g) – ^{1}H NMR (600 MHz)

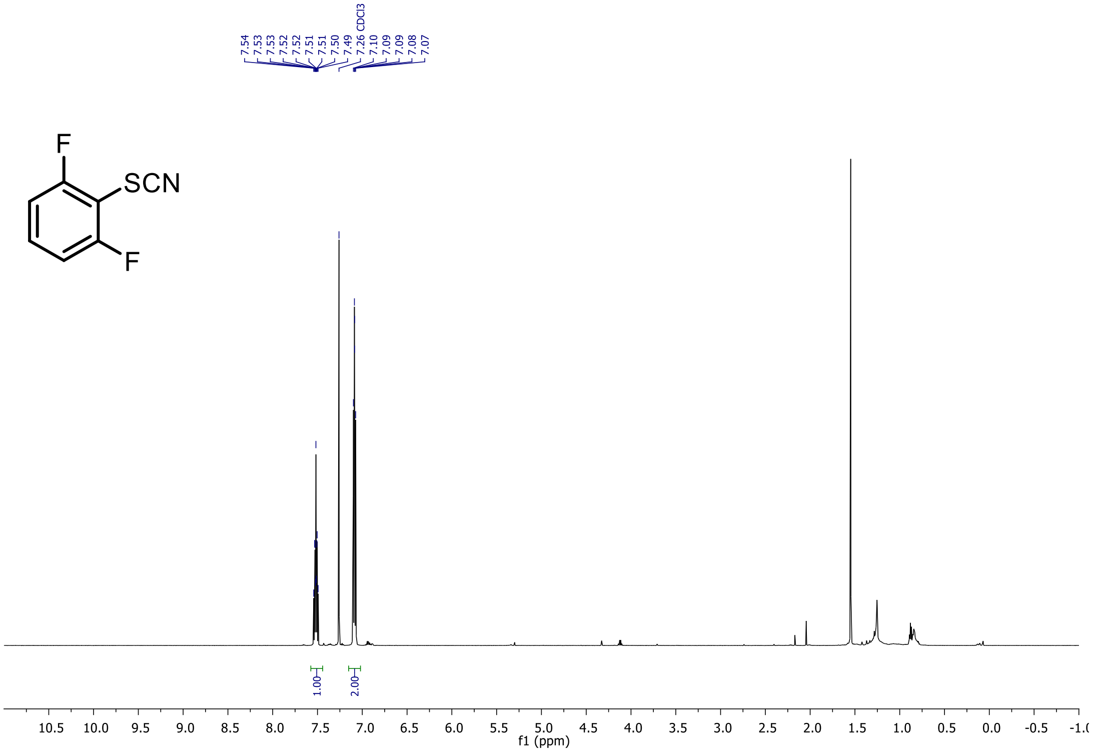

1,3-difluoro-2-thiocyanatobenzene (2g) – ^{13}C NMR (151 MHz)

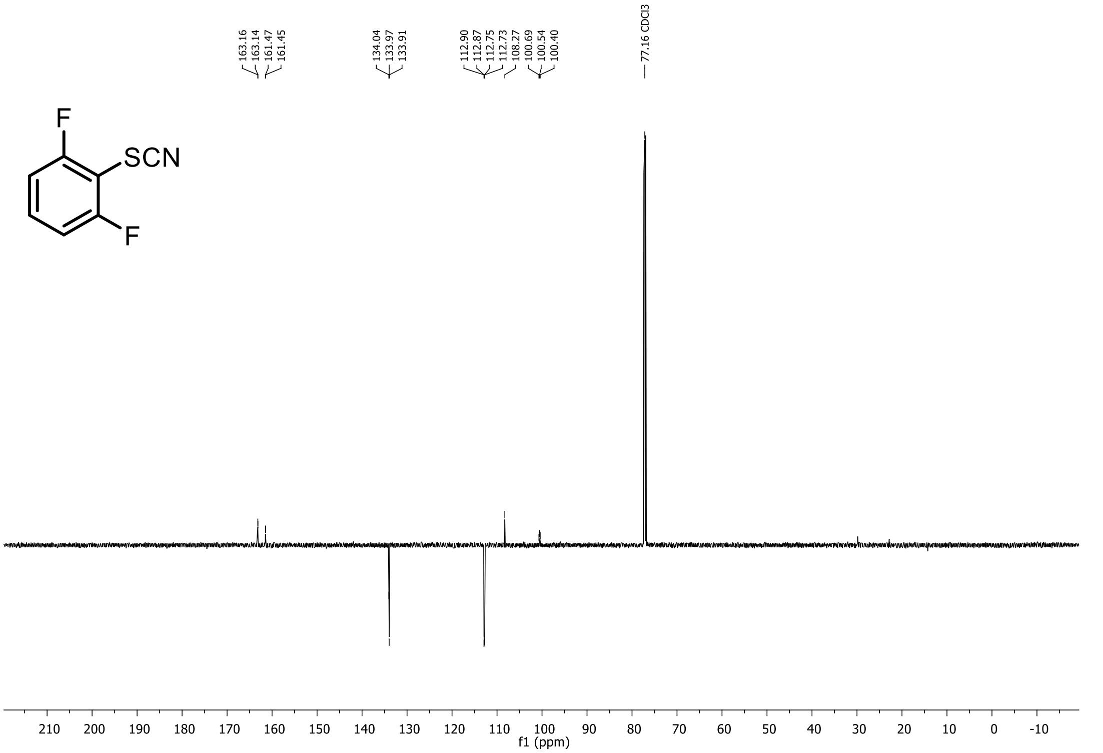

1,3-difluoro-2-thiocyanatobenzene (2g) – ^{19}F NMR (565 MHz)

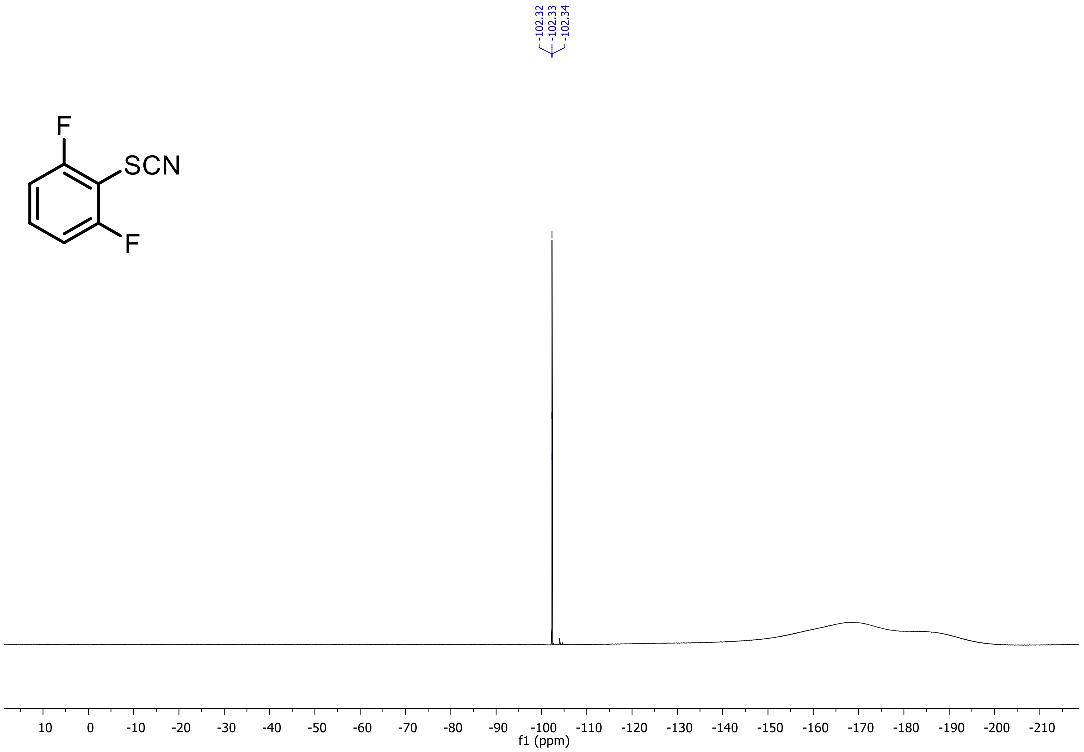

2-thiocyanatonaphthalene (2h) – ^{1}H NMR (600 MHz)

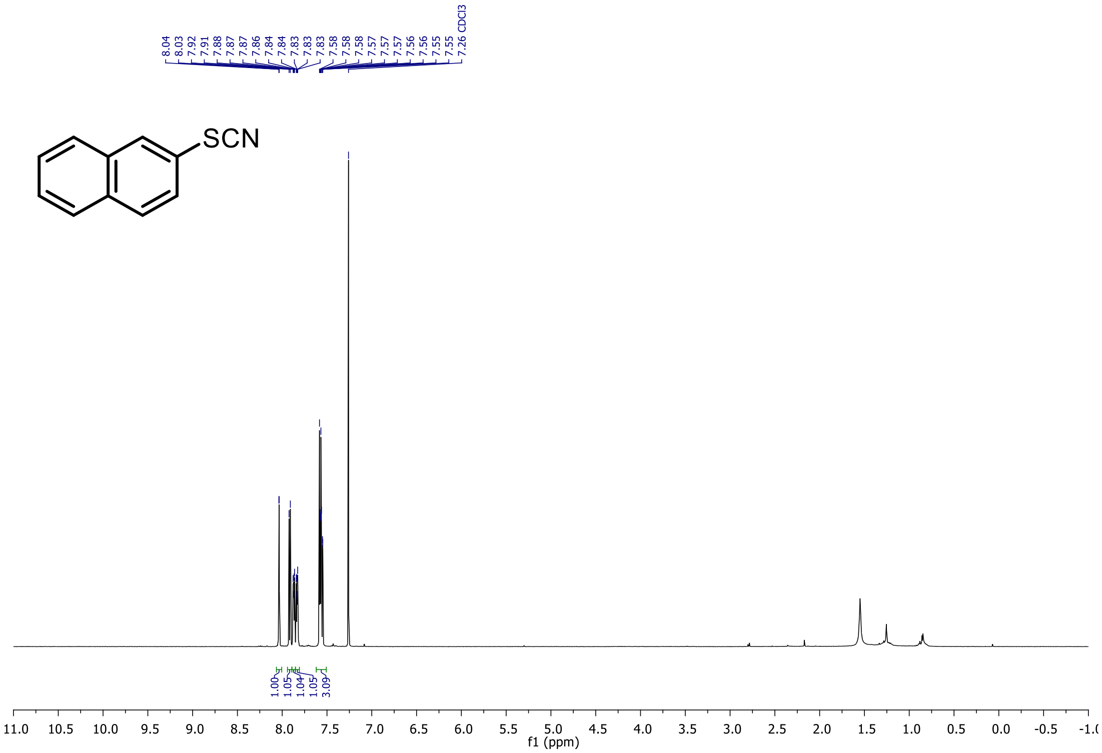

2-thiocyanatonaphthalene (2h) – ^{13}C NMR (151 MHz)

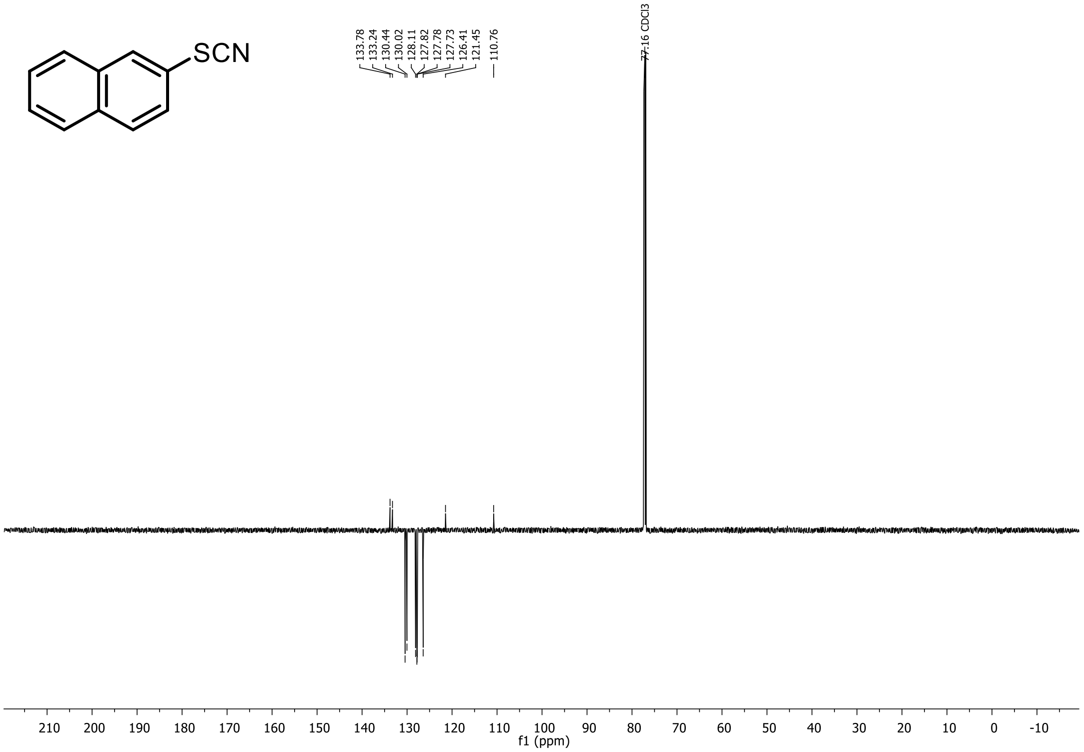

1-methoxy-2-thiocyanatobenzene (2i) – ^{1}H NMR (600 MHz)

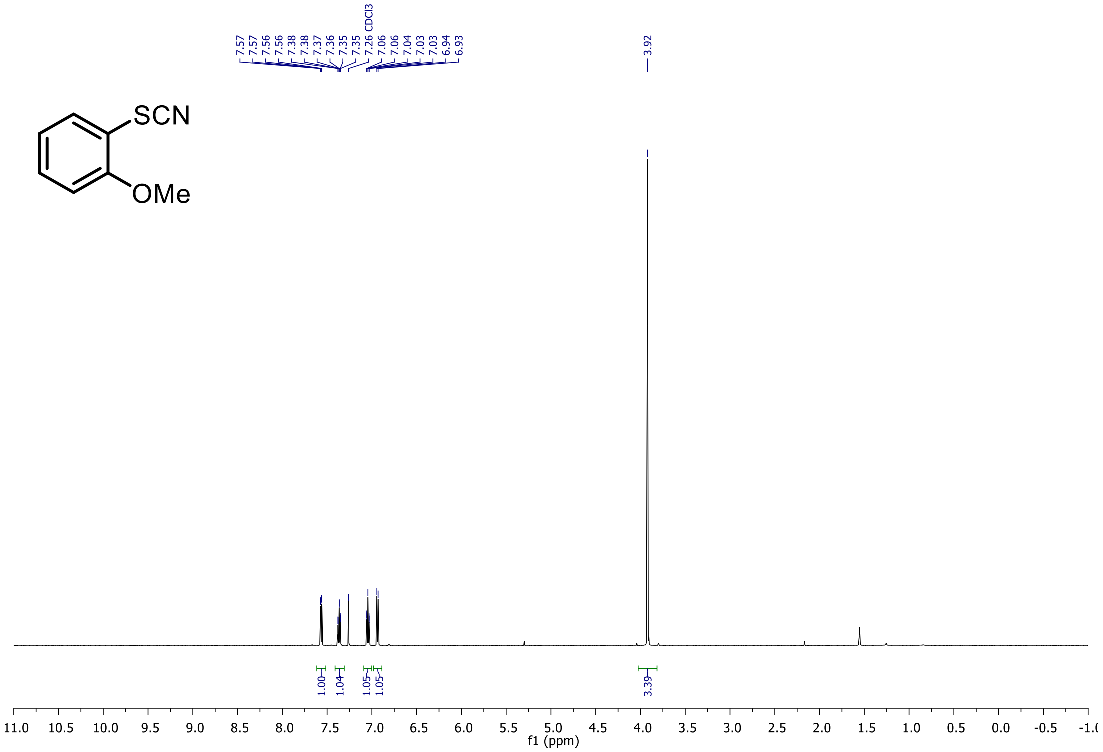

1-methoxy-2-thiocyanatobenzene (2i) – ^{13}C NMR (151 MHz)

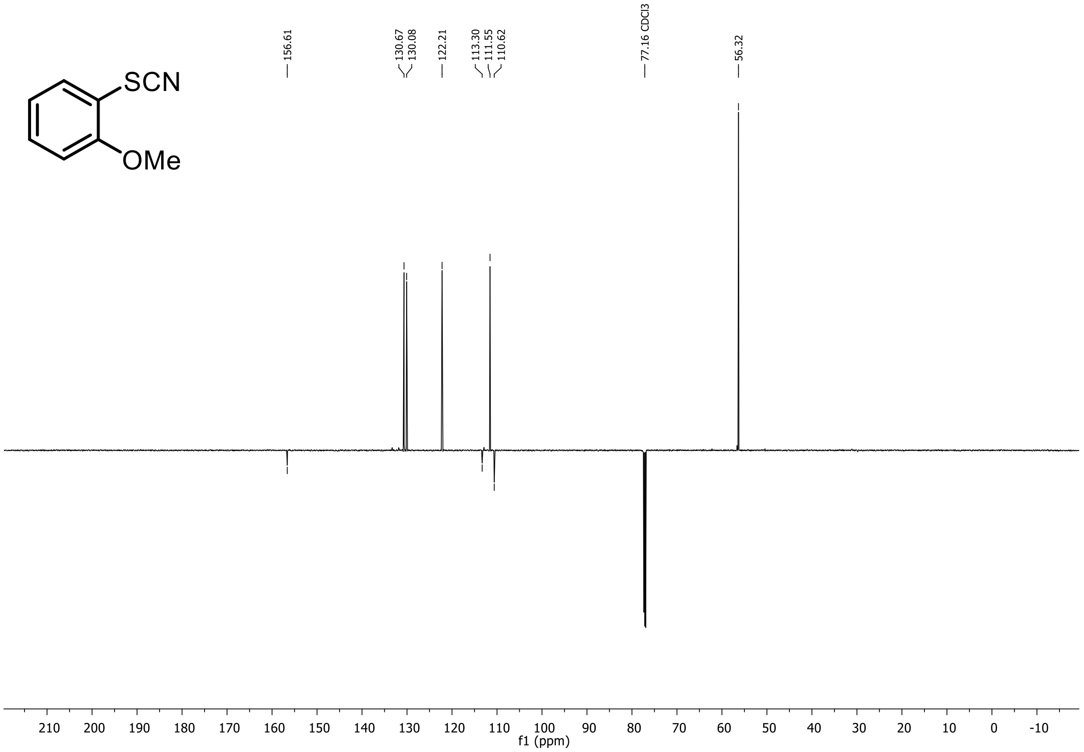

1,3-dimethoxy-2-thiocyanatobenzene (2j) – ^{1}H NMR (400 MHz)

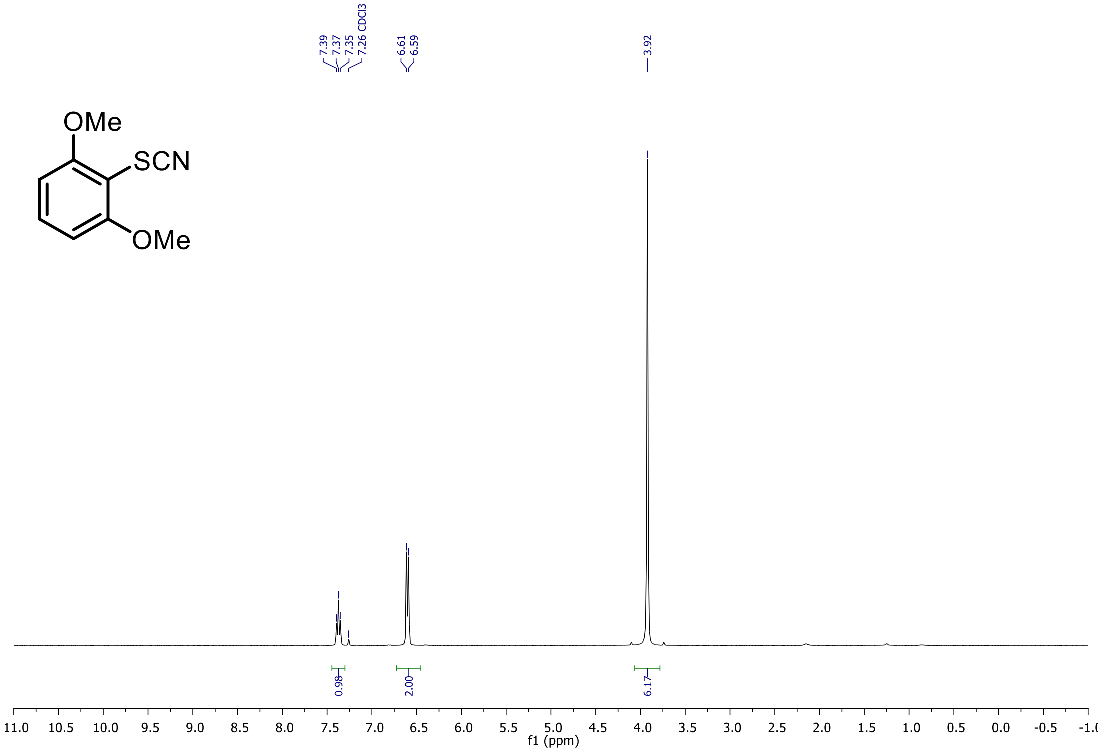

1,3-dimethoxy-2-thiocyanatobenzene (2j) – ^{13}C NMR (101 MHz)

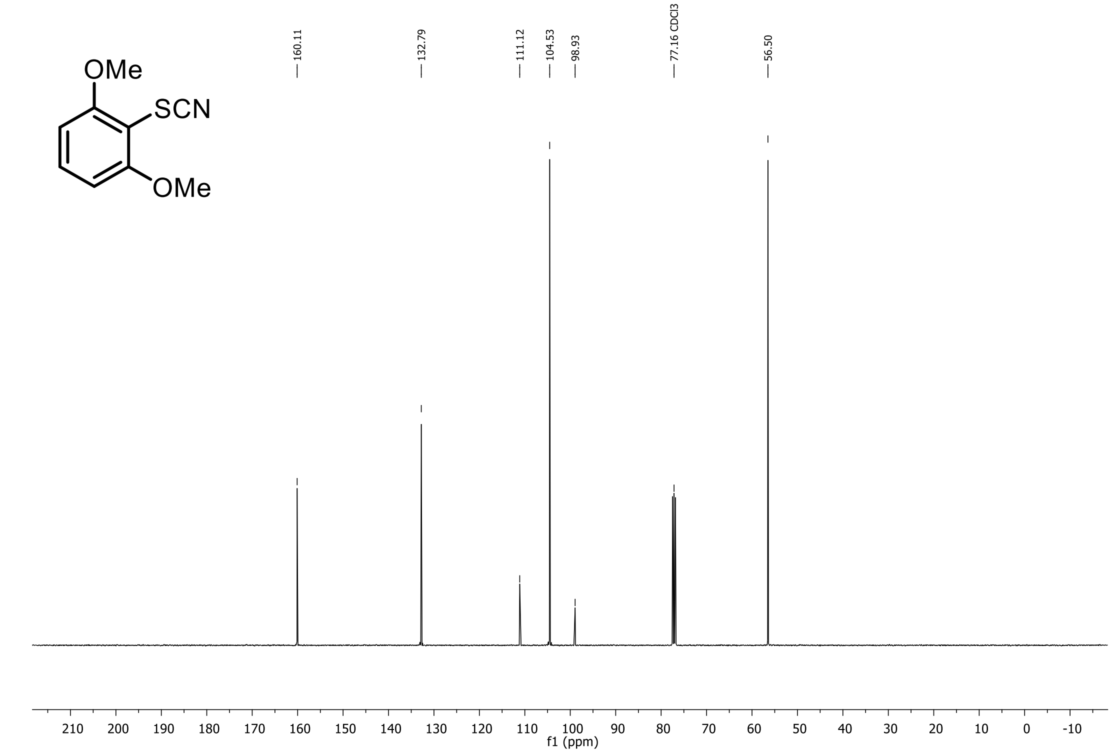

1-nitro-3-thiocyanatobenzene (2k) – ^{1}H NMR (600 MHz)

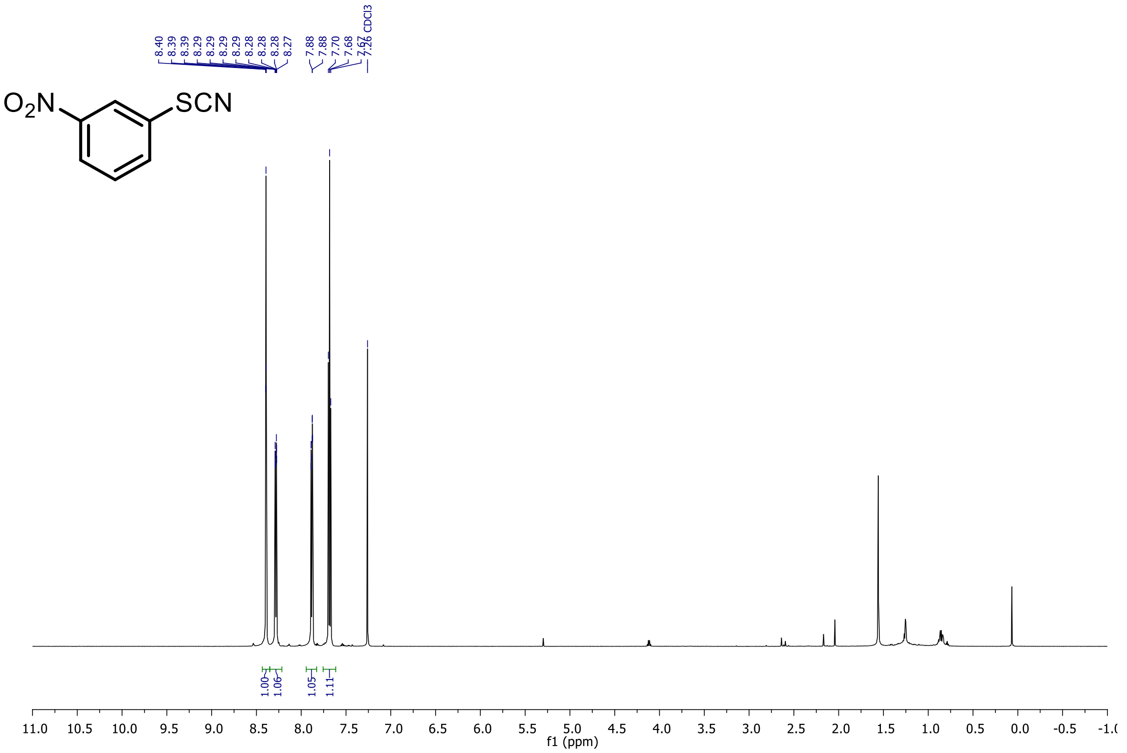

1-nitro-3-thiocyanatobenzene (2k) – ^{13}C NMR (151 MHz)

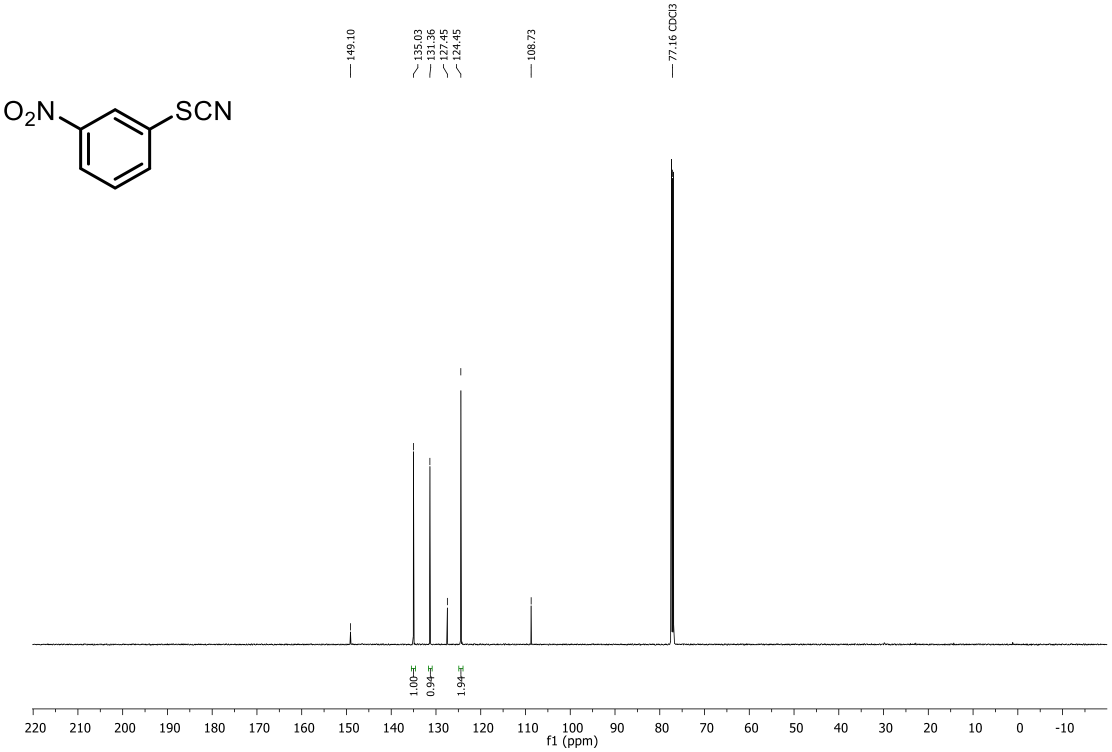

methyl 3-methoxy-4-thiocyanato-2-naphthoate (2l) – ^{1}H NMR (400 MHz)

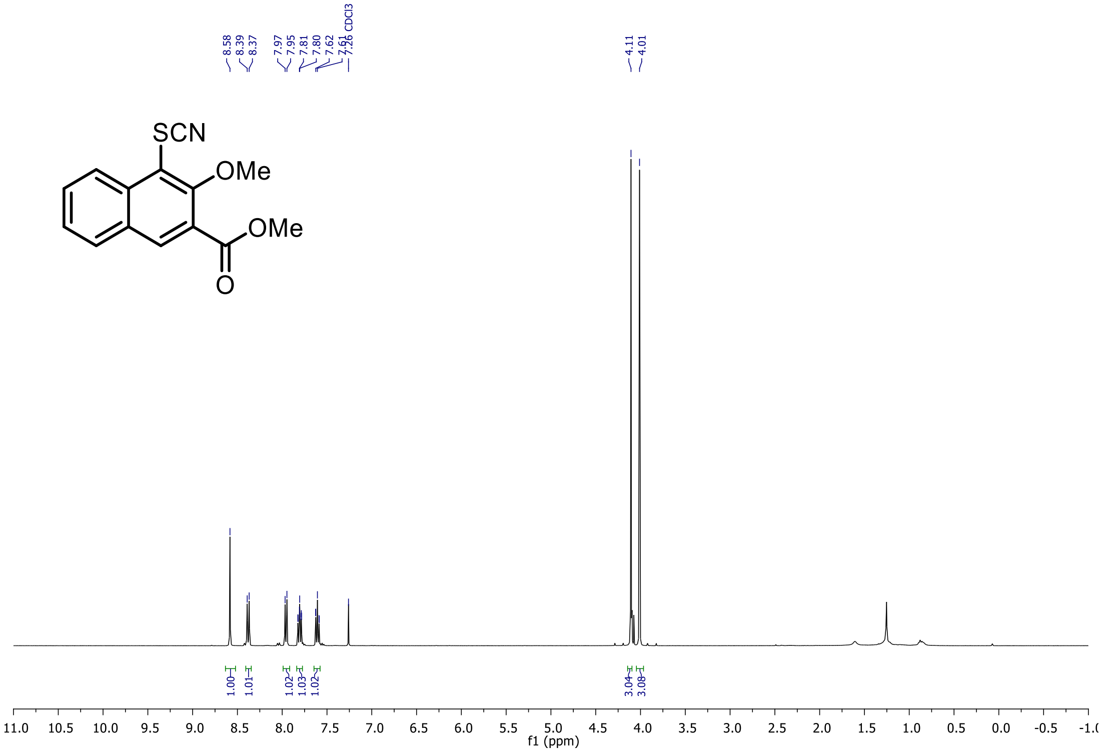

methyl 3-methoxy-4-thiocyanato-2-naphthoate (2l) – ^{13}C NMR (101 MHz)

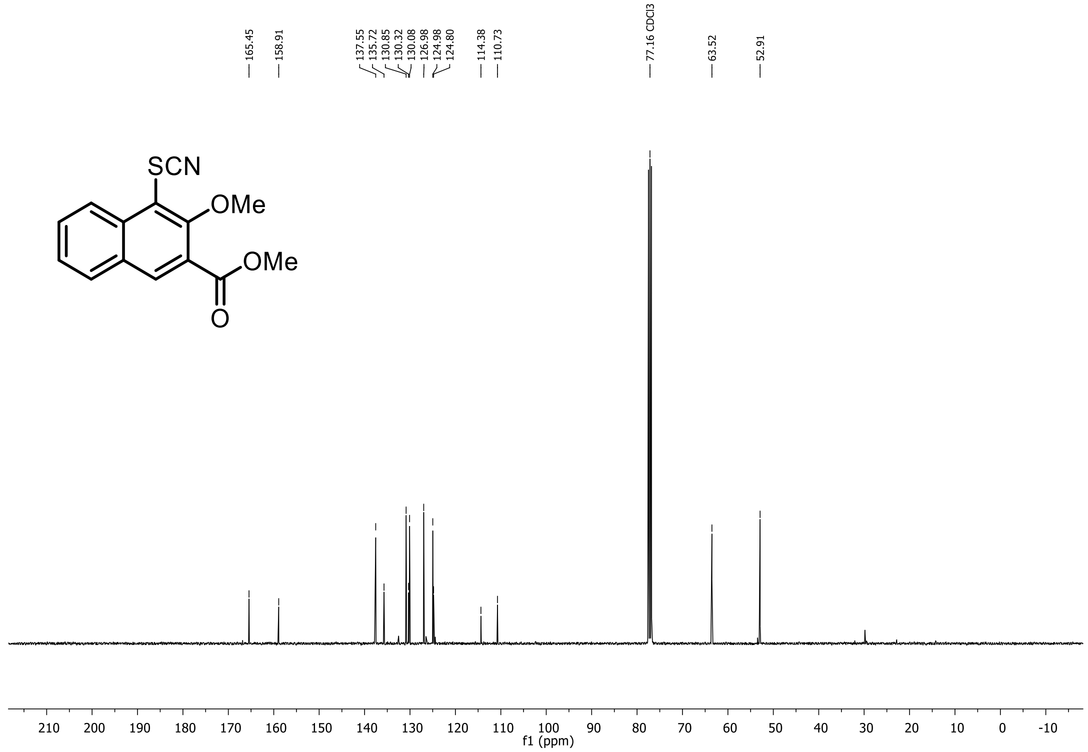

1-thiocyanatooctane (2m) – ^{1}H NMR (600 MHz)

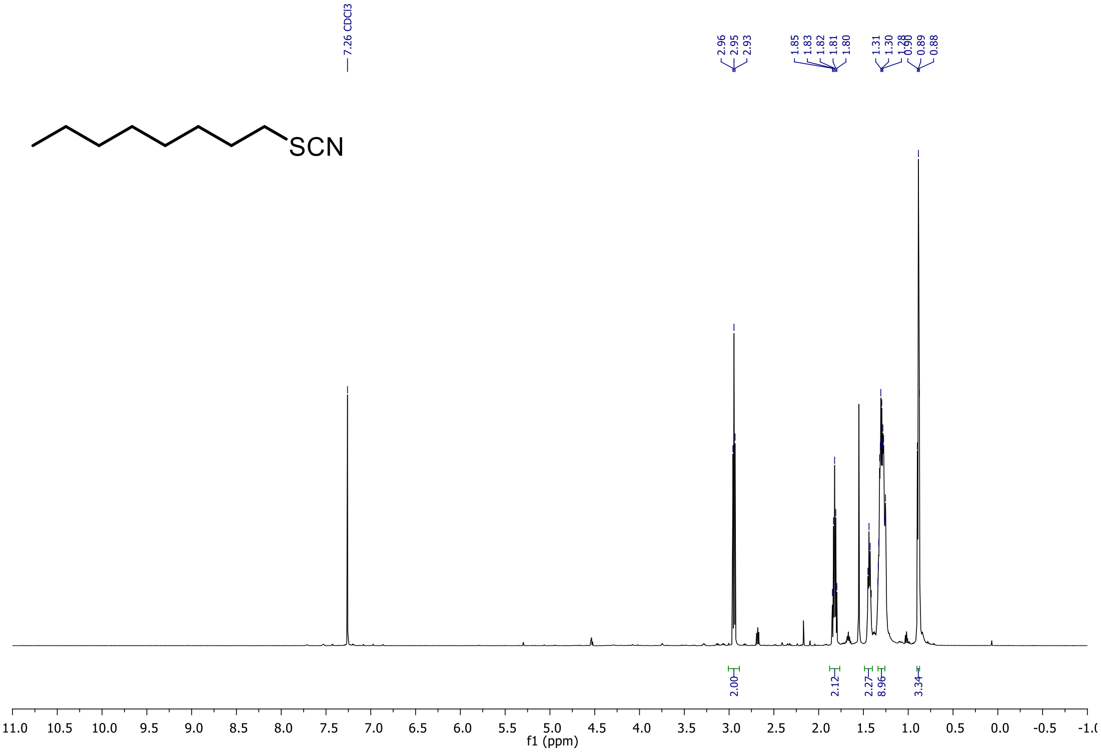

1-thiocyanatooctane (2m) – ^{13}C NMR (151 MHz)

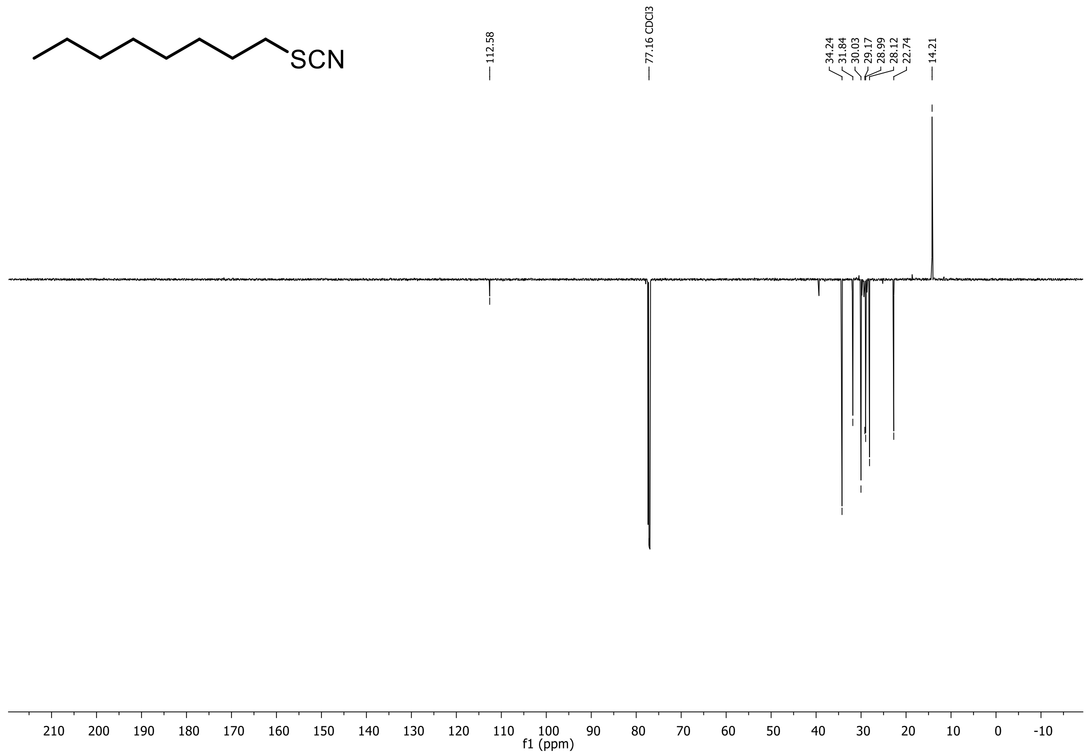

3,5-dimethyl-4-thiocyanatophenyl methylcarbamate (2n) – ^{1}H NMR (600 MHz)

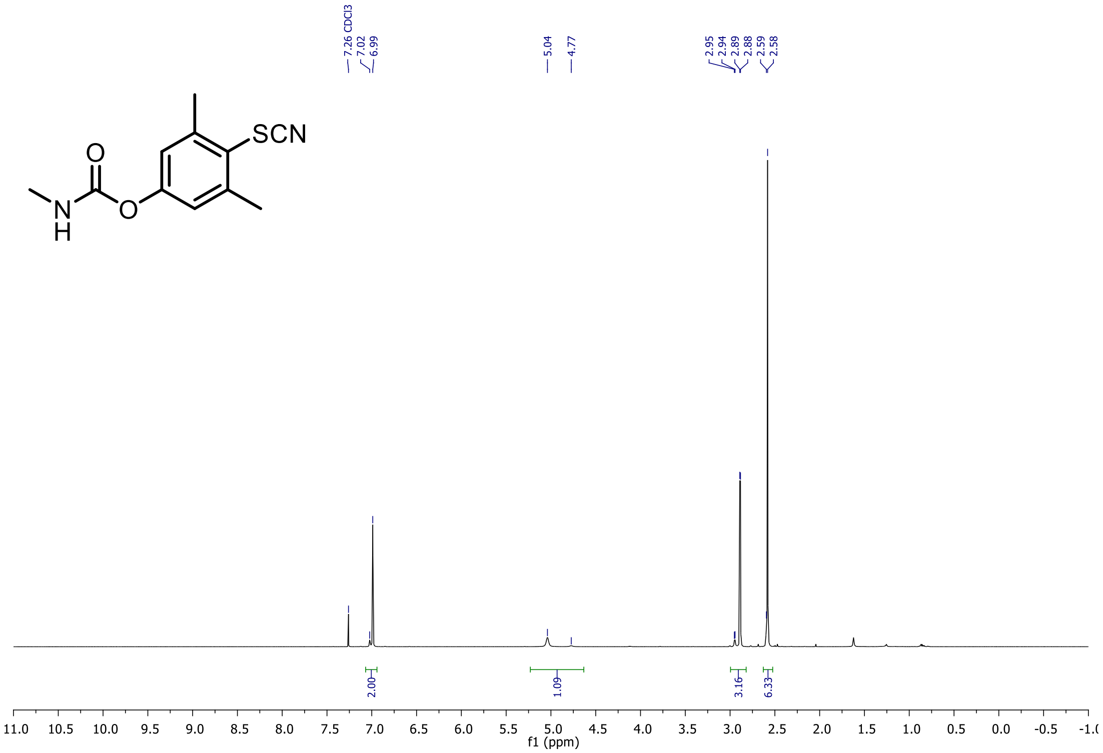

3,5-dimethyl-4-thiocyanatophenyl methylcarbamate (2n) – ^{13}C NMR (151 MHz)

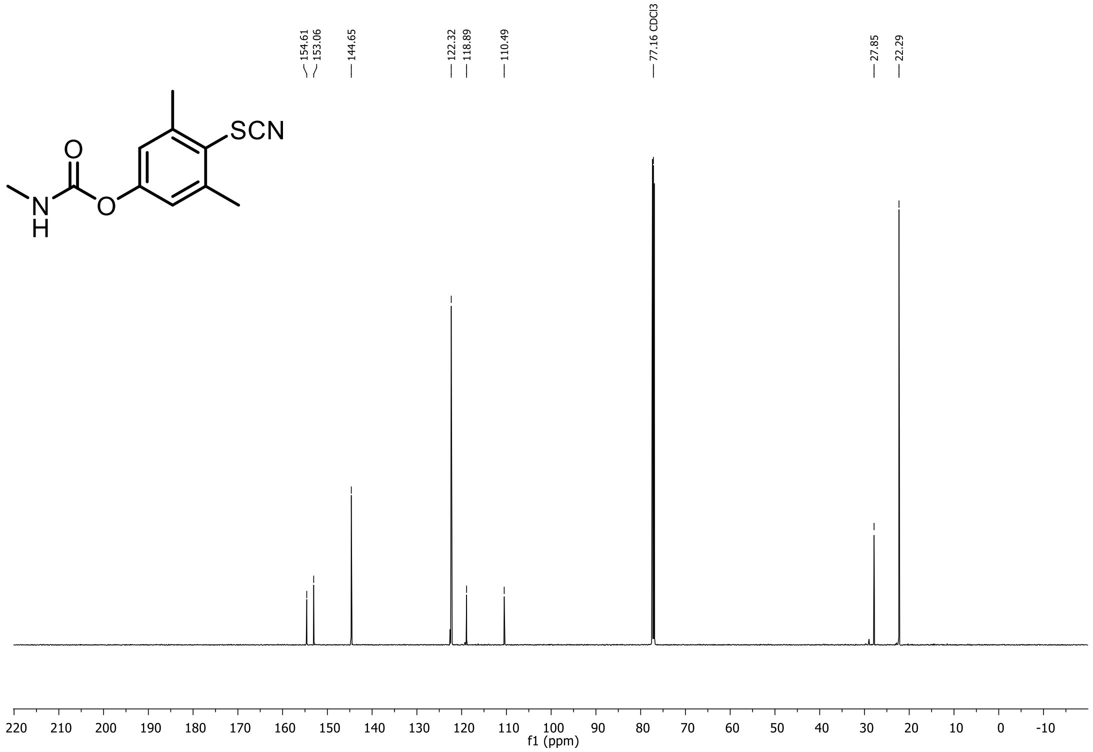

(2-thiocyanatoethyl)benzene (1o) – ^{1}H NMR (600 MHz)

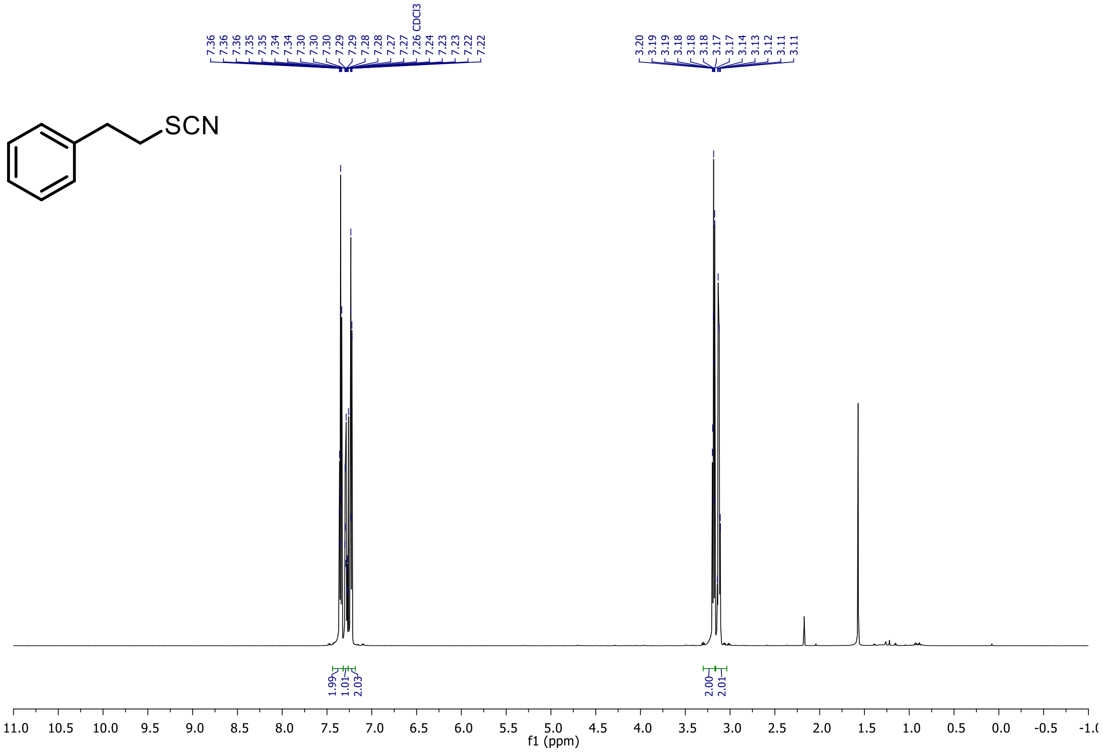

(2-thiocyanatoethyl)benzene (1o) – ^{13}C NMR (151 MHz)

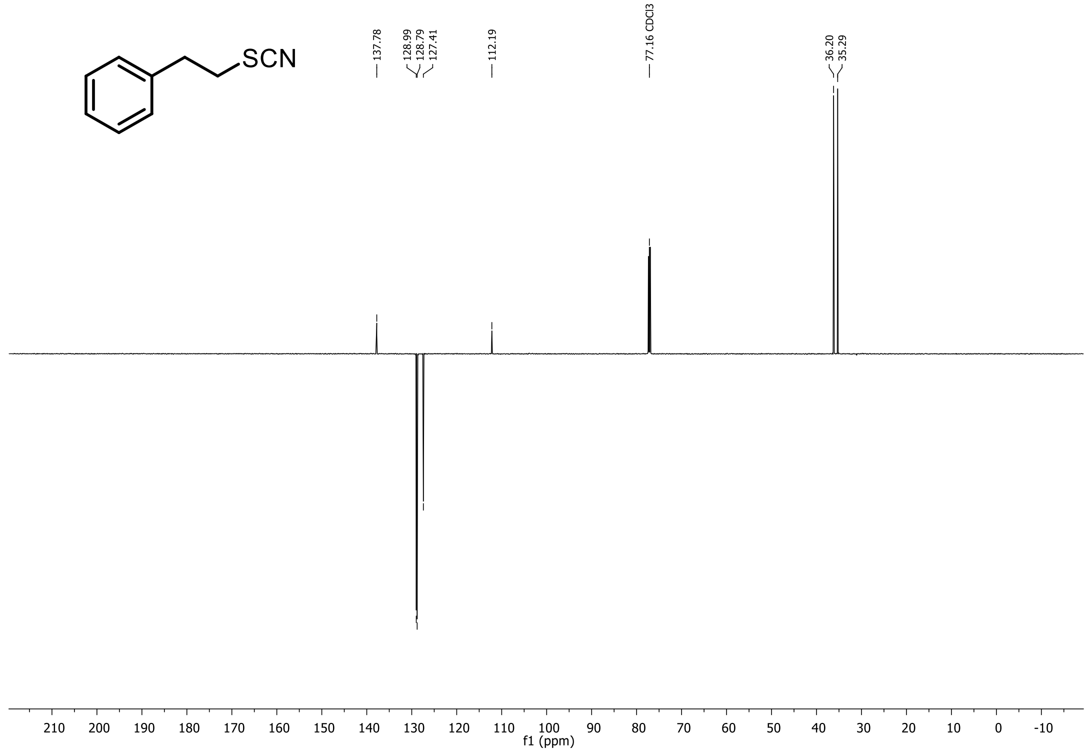

3-(2-thiocyanatophenyl)propyl trifluoromethanesulfonate (2p) – ^{1}H NMR (600 MHz)

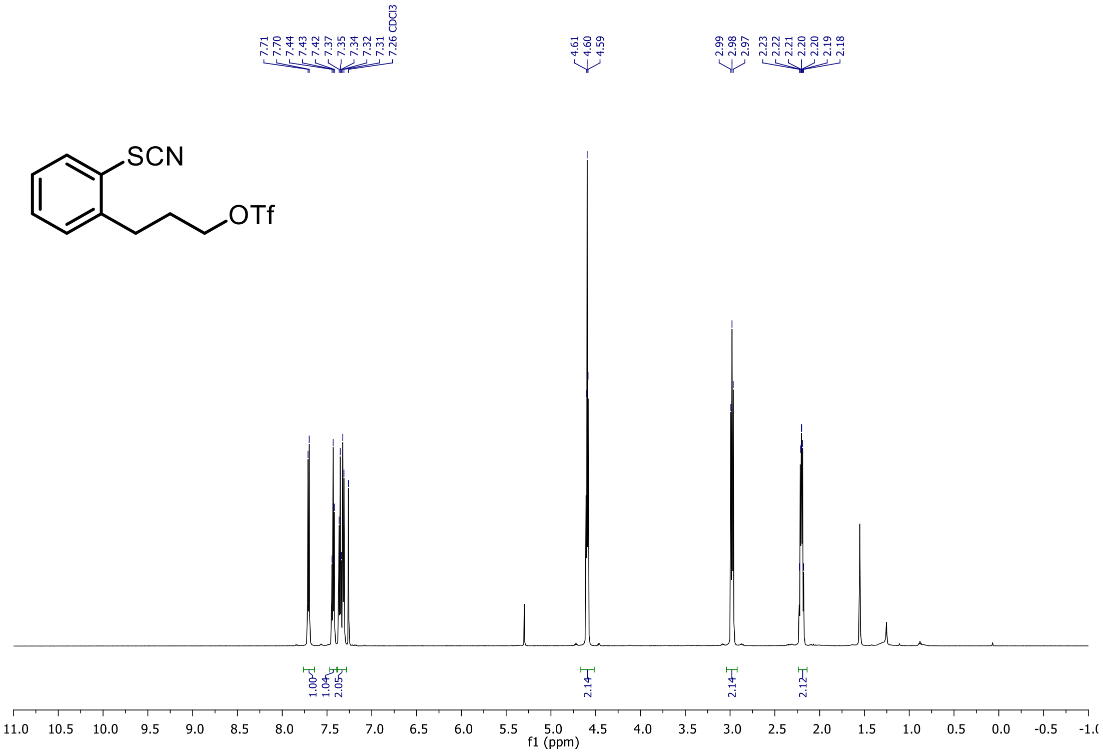

3-(2-thiocyanatophenyl)propyl trifluoromethanesulfonate (2p) – ^{13}C NMR (151 MHz)

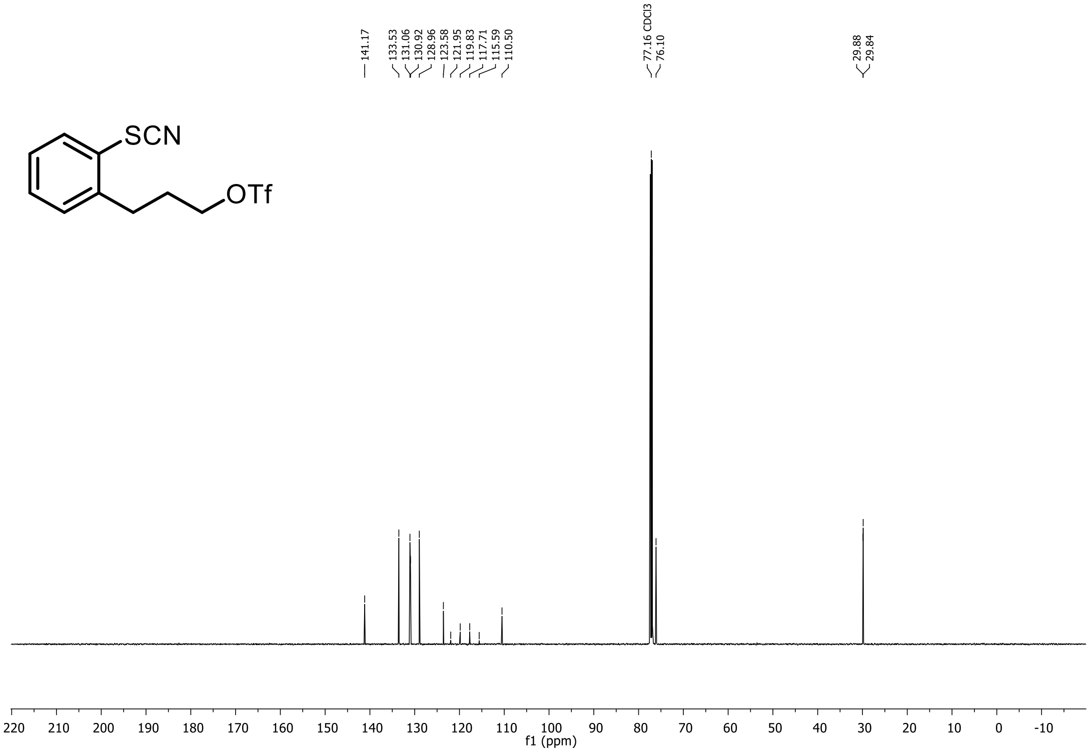

3-(2-thiocyanatophenyl)propyl trifluoromethanesulfonate (2p) – ^{19}F NMR (565 MHz)

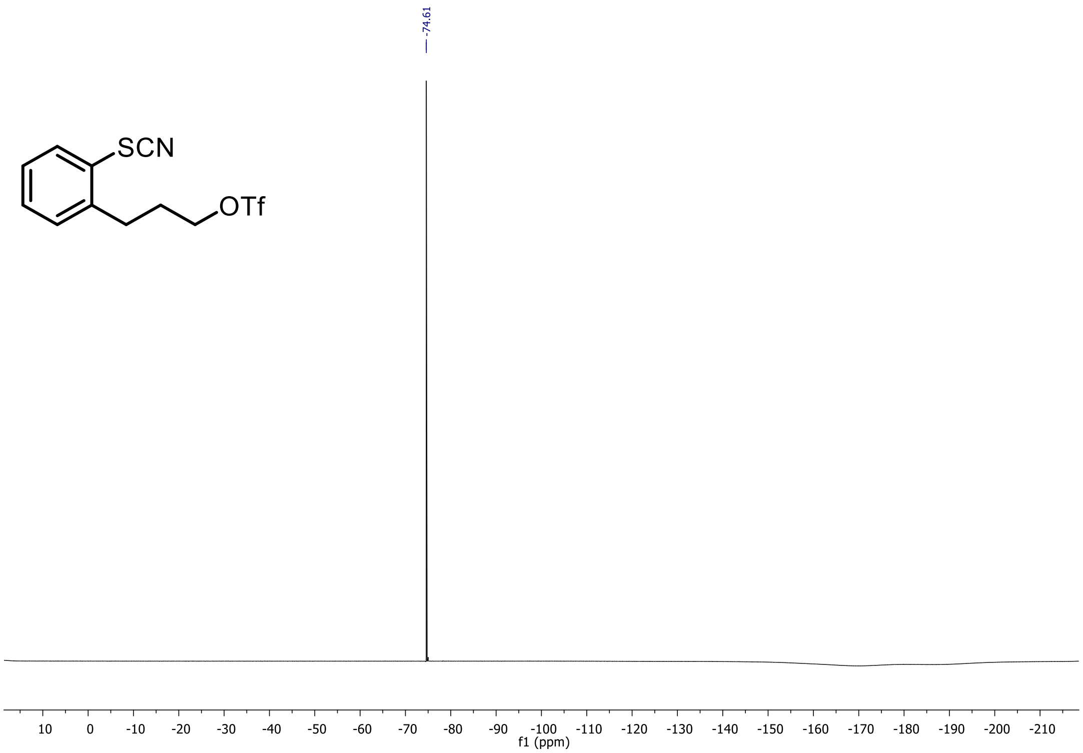

hex-1-yn-1-yl(naphthalen-2-yl)sulfane (3) – ^{1}H NMR (600 MHz)

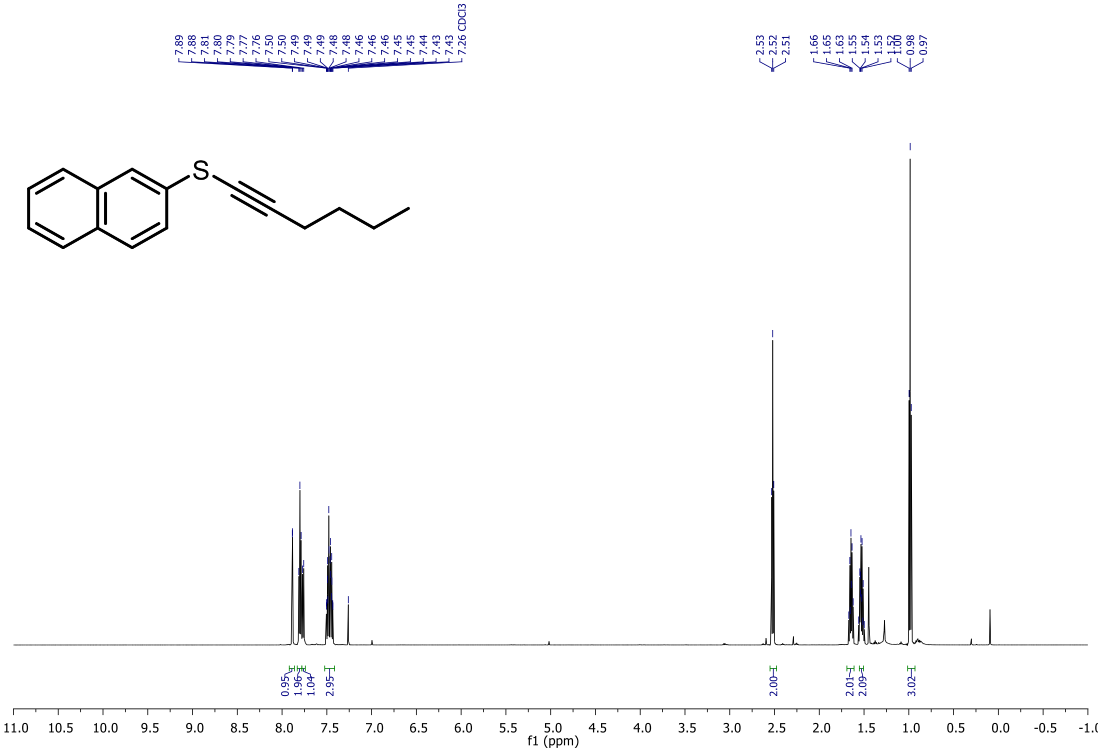

hex-1-yn-1-yl(naphthalen-2-yl)sulfane (3) – ^{13}C NMR (151 MHz)

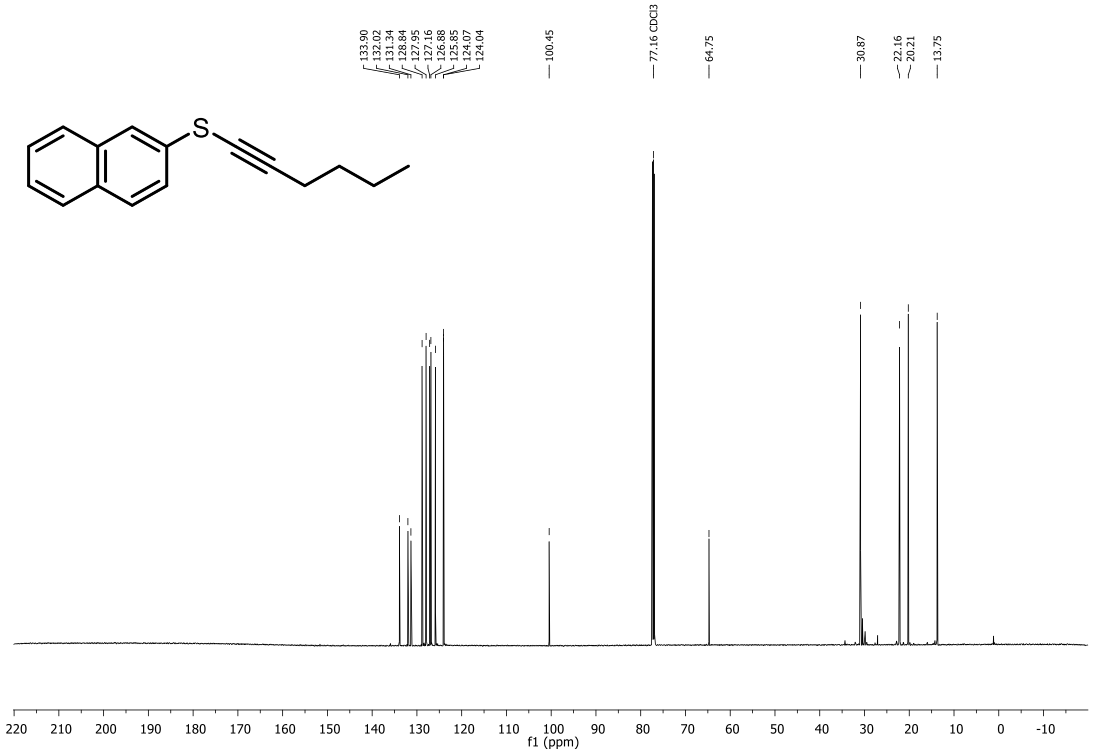

naphthalen-2-yl(trifluoromethyl)sulfane (4) – ^{1}H NMR (600 MHz)

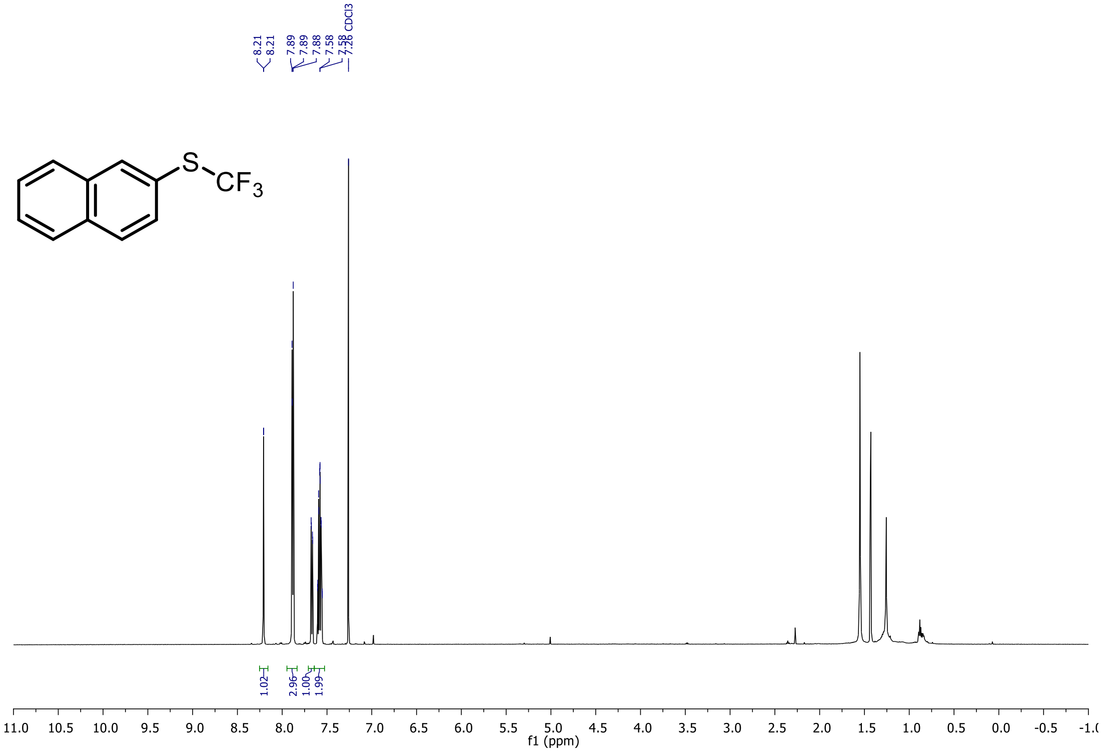

naphthalen-2-yl(trifluoromethyl)sulfane (4) – ^{13}C NMR (151 MHz)

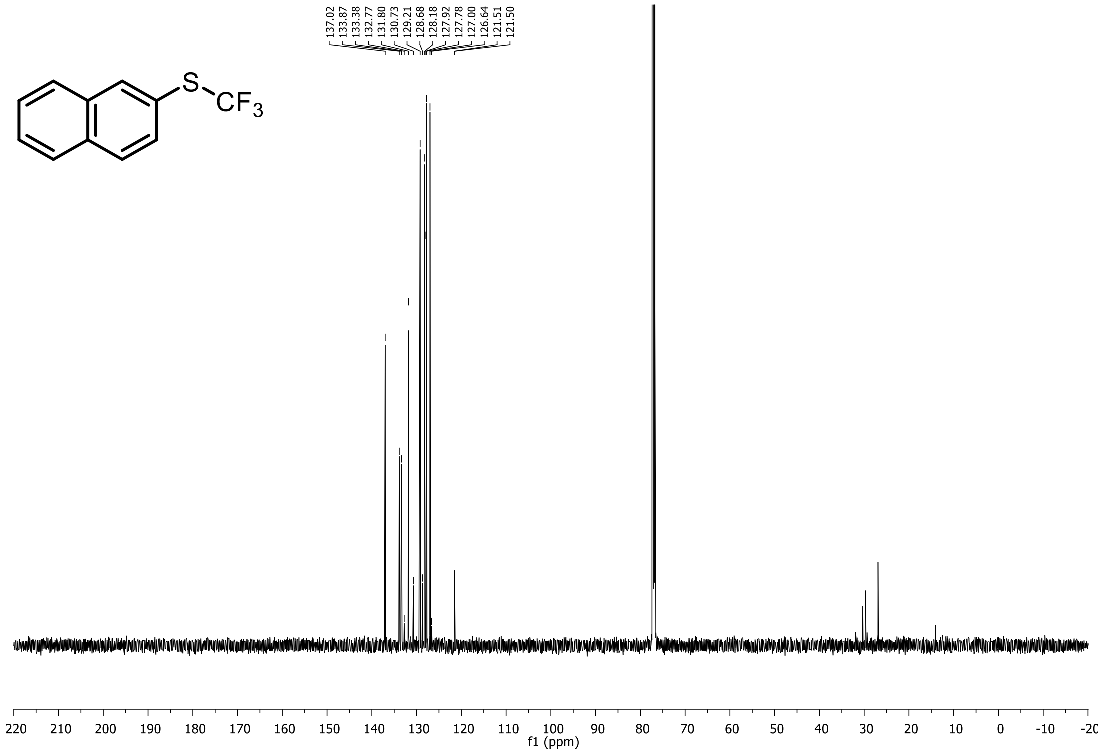

naphthalen-2-yl(trifluoromethyl)sulfane (4) – ^{19}F NMR (565 MHz)

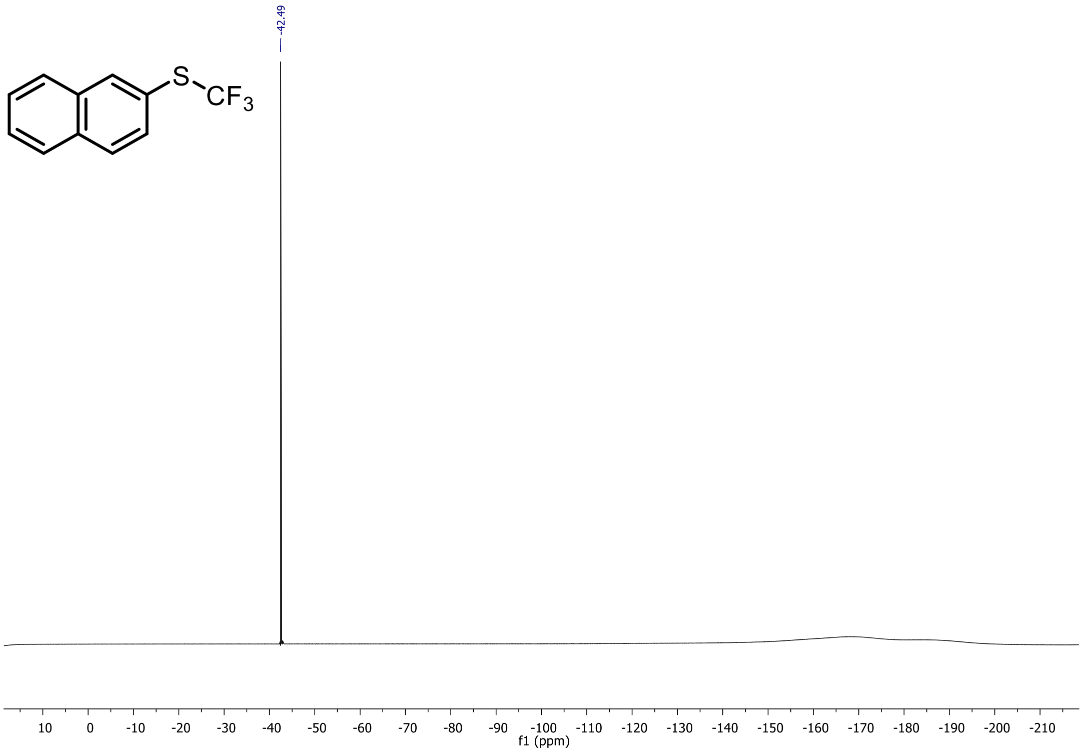

naphthalene-2-sulfonyl cyanide (5) – ^{1}H NMR (400 MHz)

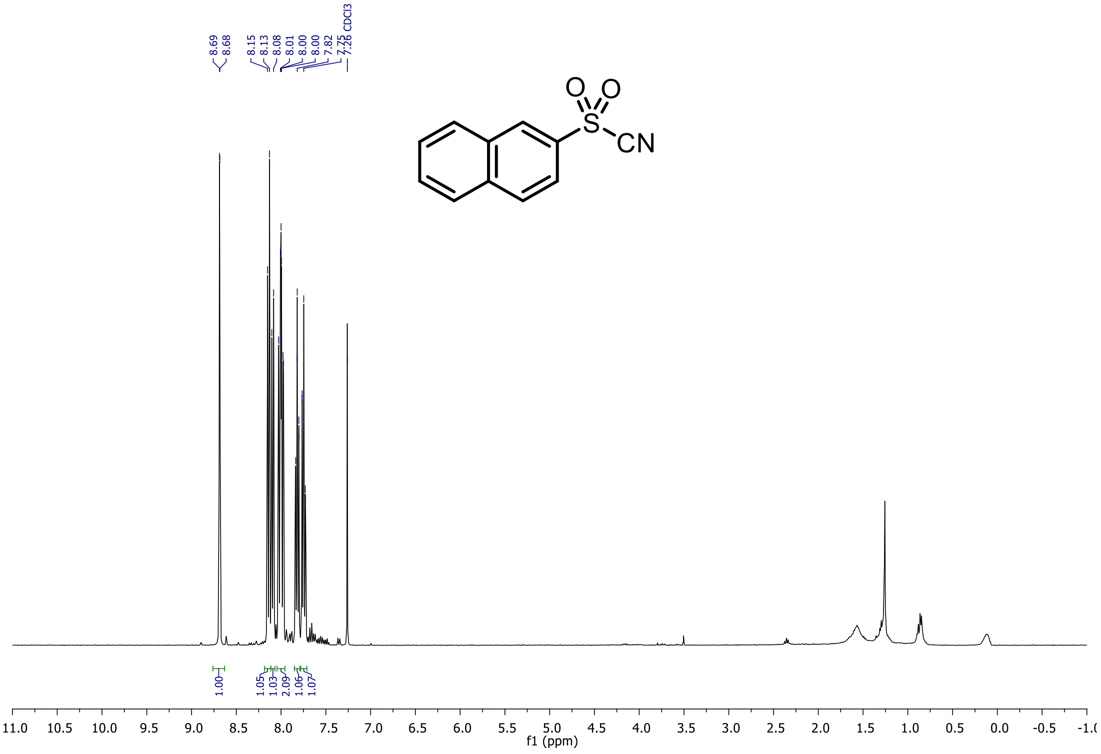

naphthalene-2-sulfonyl cyanide (5) – ^{13}C NMR (101 MHz)

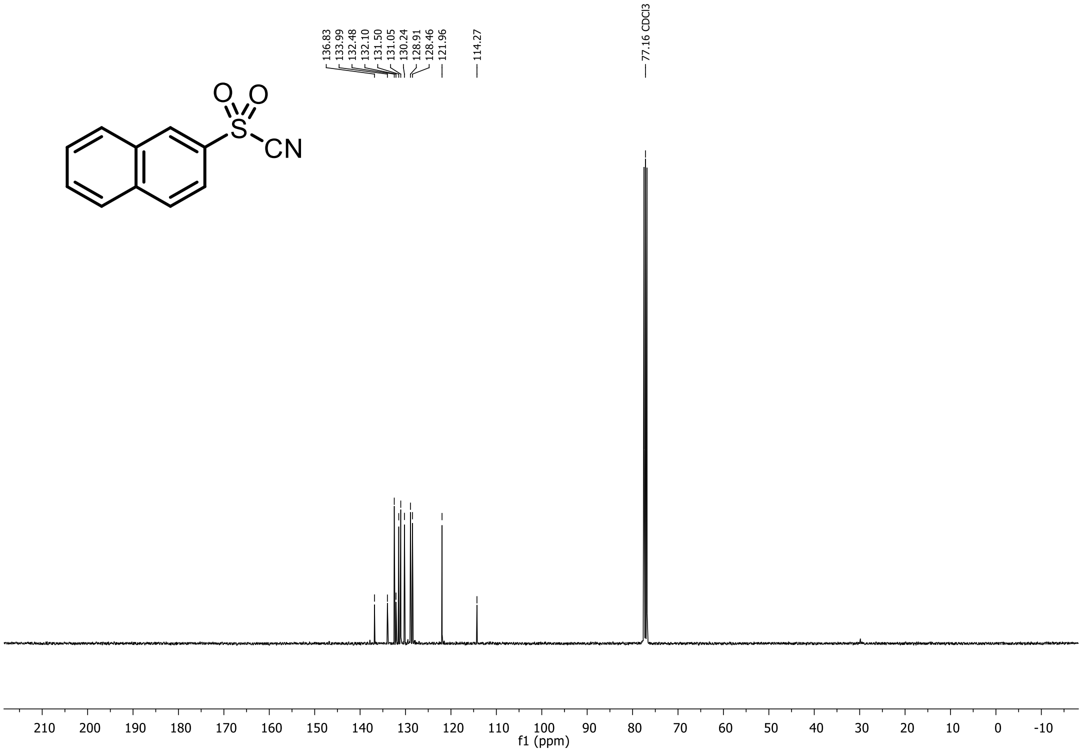

4. References

(1) Thomas E. Dueber, P. J. S. Preparation of Vinyl Trifluoromethanesulfonates: 3-Methyl-2-Buten-2-Yl Triflate. *Org. Synth.* **1974**, *54* (September), 79. https://doi.org/10.15227/orgsyn.054.0079.

(2) Kaiser, D.; Veiros, L. F.; Maulide, N. Brønsted Acid-Mediated Hydrative Arylation of Unactivated Alkynes. *Chem. - A Eur. J.* **2016**, *22* (14), 4727–4732. https://doi.org/10.1002/chem.201600432.

(3) Kaldre, D.; Maryasin, B.; Kaiser, D.; Gajsek, O.; González, L.; Maulide, N. An Asymmetric Redox Arylation: Chirality Transfer from Sulfur to Carbon through a Sulfonium [3,3]-Sigmatropic Rearrangement. *Angew. Chemie - Int. Ed.* **2017**, *56* (8), 2212–2215. https://doi.org/10.1002/anie.201610105.

(4) Maryasin, B.; Kaldre, D.; Galaverna, R.; Klose, I.; Ruider, S.; Drescher, M.; Kählig, H.; González, L.; Eberlin, M. N.; Jurberg, I. D.; Maulide, N. Unusual Mechanisms in Claisen Rearrangements: An Ionic Fragmentation Leading to a: Meta -Selective Rearrangement. *Chem. Sci.* **2018**, *9* (17), 4124–4131. https://doi.org/10.1039/c7sc04736c.

(5) Benassi, R.; Folli, U.; Iarossi, D.; Mucci, A.; Schenetti, L.; Taddei, F. Conformational Study of Substituted Methyl Phenyl Sulphoxides. A Multinuclear (1H, 13C, and 17O) Approach. *J. Chem. Soc. Perkin Trans. 2* **1989**, No. 5, 517–521. https://doi.org/10.1039/p29890000517.

(6) Yanagi, T.; Otsuka, S.; Kasuga, Y.; Fujimoto, K.; Murakami, K.; Nogi, K.; Yorimitsu, H.; Osuka, A. Metal-Free Approach to Biaryls from Phenols and Aryl Sulfoxides by Temporarily Sulfur-Tethered Regioselective C-H/C-H Coupling. *J. Am. Chem. Soc.* **2016**, *138* (44), 14582–14585. https://doi.org/10.1021/jacs.6b10278.

(7) Rioz-Martínez, A.; De Gonzalo, G.; Pazmiño, D. E. T.; Fraaije, M. W.; Gotor, V. Enzymatic Synthesis of Novel Chiral Sulfoxides Employing Baeyer-Villiger Monooxygenases. *European J. Org. Chem.* **2010**, No. 33, 6409–6416. https://doi.org/10.1002/ejoc.201000890.

(8) Nosek, V.; Míšek, J. Chemoenzymatic Deracemization of Chiral Sulfoxides. *Angew. Chemie - Int. Ed.* **2018**, *57* (31), 9849–9852. https://doi.org/10.1002/anie.201805858.

(9) Yue, H. L.; Klussmann, M. Acid-Catalyzed Oxidative Addition of Thiols to Olefins and Alkynes for a One-Pot Entry to Sulfoxides. *Synlett* **2016**, *27* (17), 2505–2509. https://doi.org/10.1055/s-0035-1562480.

(10) Eccles, K. S.; Morrison, R. E.; Stokes, S. P.; O'Mahony, G. E.; Hayes, J. A.; Kelly, D. M.; O'Boyle, N. M.; Fábián, L.; Moynihan, H. A.; Maguire, A. R.; Lawrence, S. E. Utilizing Sulfoxide•••iodine Halogen Bonding for Cocrystallization. *Cryst. Growth Des.* **2012**, *12* (6), 2969–2977. https://doi.org/10.1021/cg300189v.

(11) Pons, A.; Michalland, J.; Zawodny, W.; Chen, Y.; Tona, V.; Maulide, N. Vinyl Cation Stabilization by Silicon Enables a Formal Metal-Free α-Arylation of Alkyl Ketones. *Angew. Chemie - Int. Ed.* **2019**, *58* (48), 17303–17306. https://doi.org/10.1002/anie.201909381.

(12) Hanson, P.; Hendrickx, R. A. A. J.; Smith, J. R. L. An Investigation by Means of Correlation Analysis into the Mechanisms of Oxidation of Aryl Methyl Sulfides and Sulfoxides by Dimethyldioxirane in Various Solvents. *Org. Biomol. Chem.* **2008**, *6* (4), 745–761. https://doi.org/10.1039/b714707d.

(13) Koziakov, D.; Majek, M.; Jacobi Von Wangelin, A. Metal-Free Radical Thiolations Mediated by Very Weak Bases. *Org. Biomol. Chem.* **2016**, *14* (48), 11347–11352. https://doi.org/10.1039/c6ob02276f.

(14) Son, J. K.; Woodard, R. W. Stereochemical Mechanism of Iodoacetic Acid Mediated Decomposition of L-Methionine to L-Homoserine Lactone. *J. Am. Chem. Soc.* **1989**, *111* (4), 1363–1367. https://doi.org/10.1021/ja00186a033.

(15) Newsome, W. J.; Ayad, S.; Cordova, J.; Reinheimer, E. W.; Campiglia, A. D.; Harper, J. K.; Hanson, K.; Uribe-Romo, F. J. Solid State Multicolor Emission in Substitutional Solid Solutions of Metal-Organic Frameworks. *J. Am. Chem. Soc.* **2019**, *141* (28), 11298–11303. https://doi.org/10.1021/jacs.9b05191.

(16) Sun, N.; Zhang, H.; Mo, W.; Hu, B.; Shen, Z.; Hu, X. Synthesis of Aryl Thiocyanates via Copper-Catalyzed Aerobic Oxidative Cross-Coupling between Arylboronic Acids and KSCN. *Synlett* **2013**, *24* (11), 1443–1447. https://doi.org/10.1055/s-0033-1338939.

(17) Graßl, S.; Hamze, C.; Koller, T. J.; Knochel, P. Copper-Catalyzed Electrophilic Thiolation of Organozinc Halides by Using N-Thiophthalimides Leading to Polyfunctional Thioethers. *Chem. - A Eur. J.* **2019**, *25* (15), 3752–3755. https://doi.org/10.1002/chem.201806261.

(18) Exner, B.; Bayarmagnai, B.; Jia, F.; Goossen, L. J. Iron-Catalyzed Decarboxylation of Trifluoroacetate and Its Application to the Synthesis of Trifluoromethyl Thioethers. *Chem. - A Eur. J.* **2015**, *21* (48), 17220–17223. https://doi.org/10.1002/chem.201503915.

(19) Wang, Z. H.; Ji, X. M.; Hu, M. L.; Tang, R. Y. Nitromethane as a Cyanating Reagent for the Synthesis of Thiocyanates. *Tetrahedron Lett.* **2015**, *56* (36), 5067–5070. https://doi.org/10.1016/j.tetlet.2015.07.054.

(20) Yamaguchi, K.; Sakagami, K.; Miyamoto, Y.; Jin, X.; Mizuno, N. Oxidative Nucleophilic Strategy for Synthesis of Thiocyanates and Trifluoromethyl Sulfides from Thiols. *Org. Biomol. Chem.* **2014**, *12* (45), 9200–9206. https://doi.org/10.1039/c4ob01655f.

(21) Teng, F.; Yu, J. T.; Yang, H.; Jiang, Y.; Cheng, J. Copper-Catalyzed Cyanation of Disulfides by Azobisisobutyronitrile Leading to Thiocyanates. *Chem. Commun.* **2014**, *50* (81), 12139–12141. https://doi.org/10.1039/c4cc04578e.

(22) Kim, J.-J.; Kweon, D.-H.; Cho, S.-D.; Kim, H.-K.; Jung, E.-Y.; Lee, S.-G.; Falck, J. R.; Yoon, Y.-J. 2-Cyanopyridazin-3(2H)-Ones: Effective and Chemoselective Electrophilic Cyanating Agents. *Tetrahedron* **2005**, *61* (24), 5889–5894. https://doi.org/10.1016/J.TET.2005.03.138.

(23) Yang, Y.; Xu, L.; Yu, S.; Liu, X.; Zhang, Y.; Vicic, D. A. Triphenylphosphine-Mediated Deoxygenative Reduction of CF_3SO_2Na and Its Application for Trifluoromethylthiolation of Aryl Iodides. *Chem. - A Eur. J.* **2016**, *22* (3), 858–863. https://doi.org/10.1002/chem.201504790.

(24) Pirenne, V.; Kurtay, G.; Voci, S.; Bouffier, L.; Sojic, N.; Robert, F.; Bassani, D. M.; Landais, Y. Eosin-Mediated Alkylsulfonyl Cyanation of Olefins. *Org. Lett.* **2018**, *20* (15), 4521–4525. https://doi.org/10.1021/acs.orglett.8b01828.
